# G-CSF shifts erythropoiesis from bone marrow into spleen in the setting of systemic inflammation

**DOI:** 10.26508/lsa.202000737

**Published:** 2020-11-24

**Authors:** Weiqiang Jing, Xing Guo, Fei Qin, Yue Li, Ganyu Wang, Yuxuan Bi, Xing Jin, Lihui Han, Xiaoyuan Dong, Yunxue Zhao

**Affiliations:** 1 Department of Pharmacology, School of Basic Medical Sciences, Cheeloo College of Medicine, Shandong University, Jinan, China; 2 Department of Immunology, Shandong Key Laboratory of Infection and Immunity, School of Basic Medical Sciences, Cheeloo College of Medicine, Shandong University, Jinan, China; 3 Department of Hematology, Qilu Hospital, Cheeloo College of Medicine, Shandong University, Jinan, China

## Abstract

The anemia of inflammation is related in part to abnormal erythropoiesis in bone marrow. G-CSF regulates granulopoiesis and is increased during systemic inflammation. Here, we have showed that high levels of G-CSF are associated with repression of bone marrow erythropoiesis and expansion of splenic erythropoiesis in *Escherichia coli*–infected mice and lipopolysaccharide-treated mice. Under lipopolysaccharide-induced systemic inflammatory conditions in mice, G-CSF neutralization with antibody alleviated the blockage of bone marrow erythropoiesis, prevented the enhancement of splenic erythropoiesis, ameliorated splenomegaly, and reduced the brittleness of spleen. We further demonstrated that after lipopolysaccharide treatment, *TLR4*-knockout mice display low levels of G-CSF, healthy bone marrow erythropoiesis, almost no stress erythropoiesis in the spleen, and normal size and toughness of spleen. In addition, we found HIF-mediated erythropoietin production is essential for splenic erythropoiesis in the setting of G-CSF-induced suppression of bone marrow erythropoiesis. Our findings identify G-CSF as a critical mediator of inflammation-associated erythropoiesis dysfunction in bone marrow and offer insight into the mechanism of G-CSF-induced splenic erythropoiesis. We provide experimentally significant dimension to the biology of G-CSF.

## Abstract (precis)

Granulocyte colony-stimulating factor suppresses erythropoiesis of bone marrow and promotes splenic erythropoiesis during lipopolysaccharide-induced systemic inflammation in mice.

## Introduction

G-CSF, one of the lineage-specific hematopoietic cytokines, regulates the production of neutrophilic granulocyte (1, 2, 3). In response to infection-related inflammation, G-CSF experiences an increase in endogenous production and drives emergency granulopoiesis (4, 5, 6). Recombinant human G-CSF is widely used in patients with congenital and acquired neutropenia (7, 8, 9). G-CSF is also routinely used to mobilize hematopoietic stem and progenitor cells from the bone marrow into the peripheral blood for collection and transplantation (10).

Erythropoiesis is a carefully orchestrated process that culminates in the generation of new erythrocytes to replace the old red blood cells at a constant rate. Under steady-state conditions, bone marrow is the primary site of adult erythropoiesis, but splenic erythropoiesis can occur if bone marrow erythropoiesis is suppressed (11, 12). We recently showed treatment of mice with high-dose G-CSF impairs bone marrow erythropoiesis and promotes splenic erythropoiesis (13). Infection-related inflammation suppresses bone marrow erythropoiesis and enhances splenic erythropoiesis (14, 15, 16, 17, 18). These studies and our previous findings prompted us to hypothesize G-CSF is a key molecular regulator that induces functional changes in erythropoiesis in the bone marrow and spleen under inflammatory conditions.

Systemic infections with bacteria and their related inflammation can cause splenomegaly (14, 19). Atraumatic splenic rupture associated with bacterial infection is an infrequent but lethal condition that often coexists with splenomegaly (20, 21, 22). Splenomegaly is a known complication of G-CSF administration, and G-CSF–induced spontaneous splenic rupture is a rare but potentially fatal event (23, 24, 25, 26, 27, 28). However, the pathological relevance of G-CSF in inflammation-associated splenomegaly is not well understood and there is a limited understanding of the mechanisms regulating G-CSF-induced splenomegaly and splenic rupture.

In this study, we investigated the impact of endogenous G-CSF on erythropoiesis of the bone marrow and spleen in response to LPS-induced inflammation in mice and verified the role of G-CSF in inflammation-associated splenomegaly. Erythropoiesis is mainly regulated by erythropoietin (EPO) produced principally by tubulointerstitial cells within the kidneys in adults (12, 29). Therefore, we examined the role of EPO in G-CSF–induced splenic erythropoiesis. Furthermore, we evaluated the contribution of splenic erythropoiesis to enlarged and friable spleens in G-CSF–treated mice.

## Results

### G-CSF suppresses bone marrow erythropoiesis in mice in a dose- and time-dependent and reversible manner

**Figure 1.**
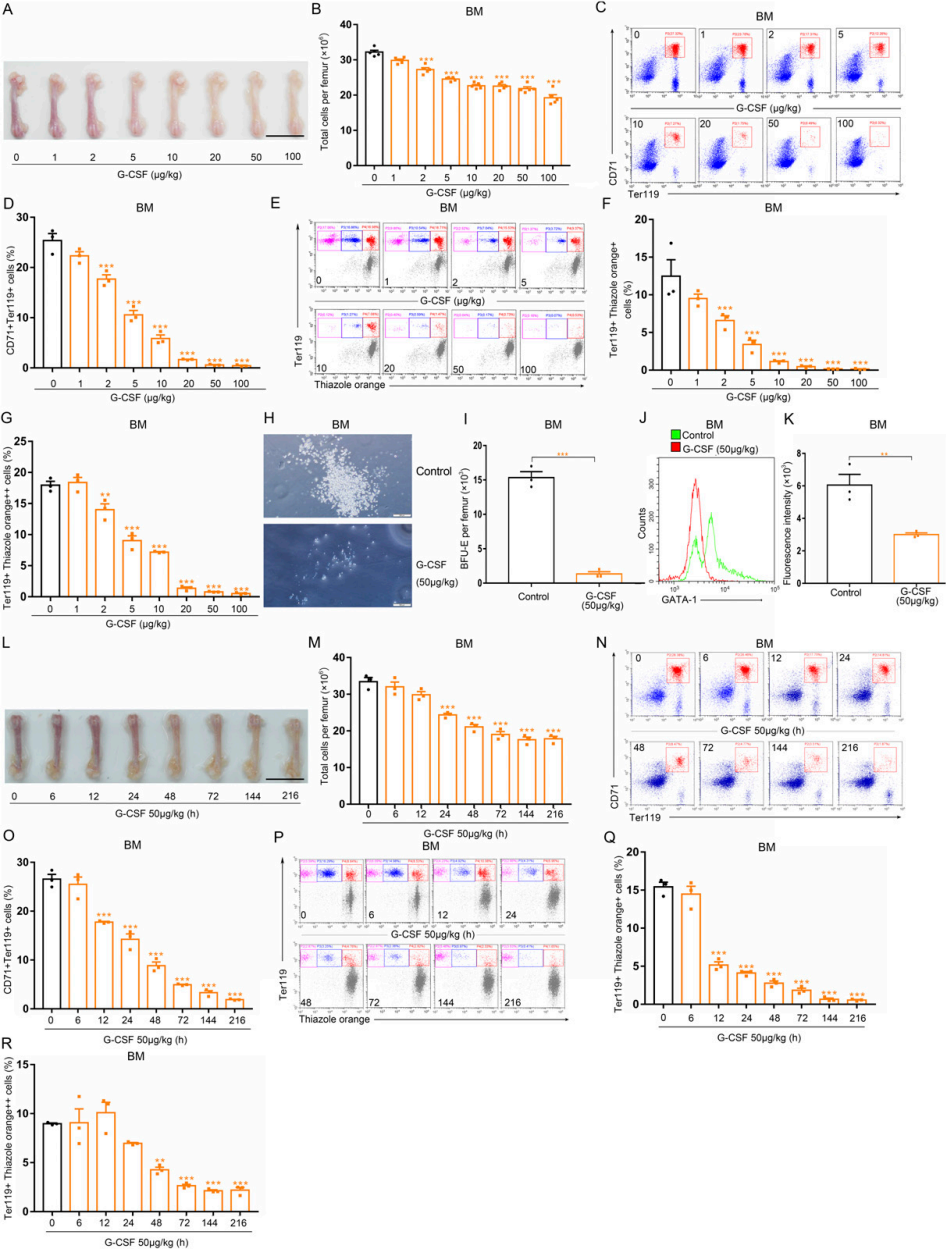
BM erythropoiesis is gradually inhibited in G-CSF–treated mice. **(A)** Representative images of femurs from control and G-CSF–treated mice (scale bar = 1 cm). **(B)** Quantification of total BM cells. **(C)** Representative flow cytometric plots of CD71^+^Ter119^+^ cells in the BMs of control and G-CSF–treated mice. **(D)** Flow cytometric quantification of CD71^+^Ter119^+^ erythroid cells in the BMs of control and G-CSF-treated mice. **(E)** Representative flow cytometric profiles of Ter119-APC and thiazole orange-stained cells from the BMs of control and G-CSF–treated mice. **(F)** Flow cytometric quantification of reticulocytes (Ter119^+^ Thiazole orange^+^) in the BM of mice. **(G)** Flow cytometric quantification of erythroblasts (Ter119^+^ Thiazole orange^++^) in the BMs of mice. **(H)** Representative fields of burst-forming unit erythroid-derived colonies in BM cultures from control and G-CSF–treated mice (×100 magnification; scale bar = 200 μm). **(I)** Quantification of the number of burst-forming unit erythroid-derived colonies in BM cultures from control and G-CSF treated mice. **(J, K)** Flow cytometric analyses of GATA-1 expression in BM cells from control and G-CSF–treated mice. **(J)** Representative histograms. **(K)** Mean fluorescence intensities were graphed. **(L)** Representative images of femurs from control and G-CSF–treated mice (scale bar = 1 cm). **(M)** Quantification of total BM cells. **(N)** Representative flow cytometric plots of CD71^+^Ter119^+^ BM cells for each indicated group. **(O)** Flow cytometric quantification of CD71^+^Ter119^+^ erythroid cells in the BMs of control and G-CSF–treated mice. **(P)** Representative flow cytometric profiles of BM cells stained with anti-Ter119 and thiazole orange for each indicated group. **(Q)** Flow cytometric quantification of reticulocytes (Ter119^+^ Thiazole orange^+^) in the BM of mice. **(R)** Flow cytometric quantification of erythroblasts (Ter119^+^ Thiazole orange^++^) in the BMs of mice. n = 5/group (A, B), n = 3/group (C, D, E, F, G, H, I, J, K, L, M, N, O, P, Q, R). **P < 0.01, ***P < 0.001 versus controls. Data are from one experiment representative of three experiments.

To characterize the effect of G-CSF on erythropoiesis globally, we first performed a detailed analysis of the relationship between G-CSF dosage and alteration in erythropoiesis in the bone marrow of mice. G-CSF (1, 2, 5, 10, 20, 50, or 100 μg/kg) was subcutaneously administered twice daily to male C57BL/6 mice for 9 d. The mouse femurs became progressively paler in response to the gradually increasing doses of G-CSF (Fig 1A). Furthermore, cell-counting analysis revealed a gradual reduction in bone marrow cell number after G-CSF treatment in a dose-dependent fashion (Fig 1B). These observations suggest G-CSF treatment causes a profound alteration of hematopoiesis in bone marrow. Therefore, we performed flow cytometry to examine erythroid differentiation in the bone marrow of femurs using two markers of erythroid lineage, Ter119 and CD71. We found that the percentage of Ter119^+^CD71^+^ cells in the bone marrow of mice gradually decreased with increasing doses of G-CSF (Fig 1C and D). Thiazole orange is a fluorescent dye that is cell membrane permeable and binds to DNA and RNA (30). Reticulocyte contains a small amount of RNA and DNA. Ter119 is a cell-surface erythroid-specific marker and is expressed from proerythroblast to mature erythrocyte (13). Thiazole orange and Ter119-APC double staining can characterize the reticulocytes (Ter119^+^Thiazole orange^+^) and erythroblasts (Ter119^+^Thiazole orange^++^) in the bone marrow. We next carried out flow cytometric analysis by double staining with thiazole orange and Ter119-APC to further characterize the red blood cell precursors in G-CSF-treated mice. After treatment of mice with G-CSF, the numbers of reticulocytes and erythroblasts in the bone marrow gradually decreased (Fig 1E–G). Burst-forming unit erythroid (BFU-E) is the earliest committed erythroid progenitor (31). We found that bone marrow cells from G-CSF–treated mice (50 μg/kg) formed lower numbers of BFU-E colonies than the controls (Fig 1H and I). GATA-1, which promotes erythroid differentiation by regulating the expression of several erythroid-specific genes, is highly expressed during erythroid differentiation (32, 33). To further understand the link between G-CSF and erythropoiesis, we studied changes in GATA-1 expression in bone marrow cells in mice treated with G-CSF (50 μg/kg) using flow cytometry with specific antibodies. GATA-1 expression was decreased in the bone marrow cells of mice treated with G-CSF (Fig 1J and K).

**Figure S1.**
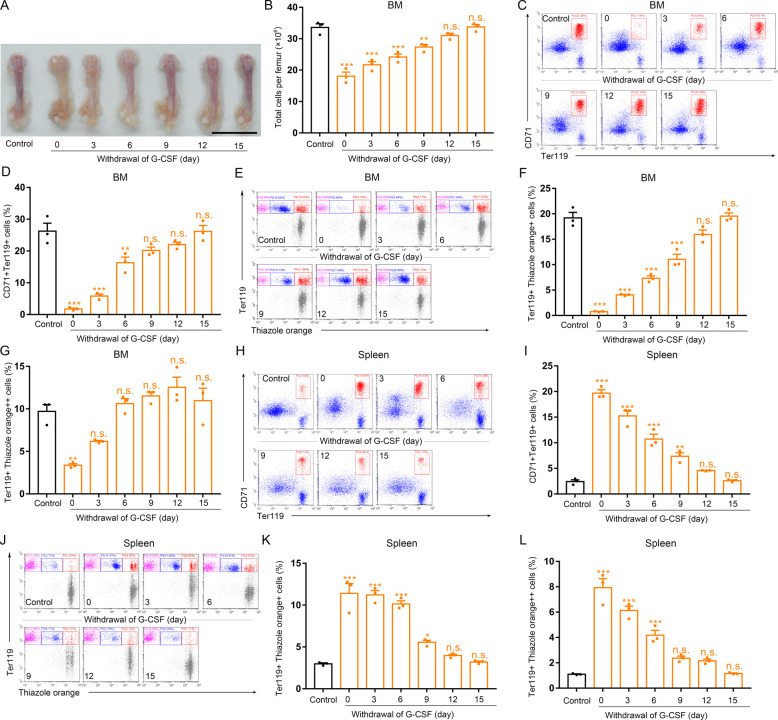
G-CSF withdrawal restores BM erythropoiesis and decreases splenic erythropoiesis in G-CSF–treated mice. G-CSF was administered to male C57BL/6 mice at 50 μg/kg for 9 d by twice-daily subcutaneous injections, and then was stopped for 3, 6, 9, 12, and 15 d. **(A)** Representative images of femurs from normal control mice, G-CSF–treated mice, and stopping treatment of mice with G-CSF (scale bar = 1 cm). **(B)** Quantification of total BM cells. **(C)** Representative flow cytometric plots of CD71^+^Ter119^+^ cells from the BMs of normal control mice, G-CSF–treated mice, and stopping treatment of mice with G-CSF. **(D)** Flow cytometric quantification of CD71^+^Ter119^+^ erythroid cells in the BMs of normal control mice, G-CSF–treated mice, and stopping treatment of mice with G-CSF. **(E)** Representative flow cytometric profiles of Ter119-APC and thiazole orange–stained cells from the BMs of control mice, G-CSF–treated mice, and stopping treatment of mice with G-CSF. **(F)** Flow cytometric quantification of reticulocytes (Ter119^+^ Thiazole orange^+^) in the BM of mice. **(G)** Flow cytometric quantification of erythroblasts (Ter119^+^ Thiazole orange^++^) in the BMs of mice. **(H)** Representative flow cytometric plots of CD71^+^Ter119^+^ cells in the spleens of control mice, G-CSF–treated mice, and stopping treatment of mice with G-CSF. **(I)** Bar graphs showing quantification of CD71^+^Ter119^+^ cells in the spleens of control mice, G-CSF–treated mice, and stopping treatment of mice with G-CSF. **(J)** Representative flow cytometric plots of Ter119-APC and thiazole orange–stained splenocytes from control mice, G-CSF–treated mice, and stopping treatment of mice with G-CSF. **(K)** Flow cytometric quantification of reticulocytes (Ter119^+^ Thiazole orange^+^) in the spleens from different treatment groups. **(L)** Flow cytometric quantification of erythroblasts (Ter119^+^ Thiazole orange^++^) in the spleens from different treatment groups. n = 3/group (A, B, C, D, E, F, G, H, I, J, K, L). *P < 0.05, **P < 0.01, ***P < 0.001 versus controls, n.s., no significance.

**Figure S2.**
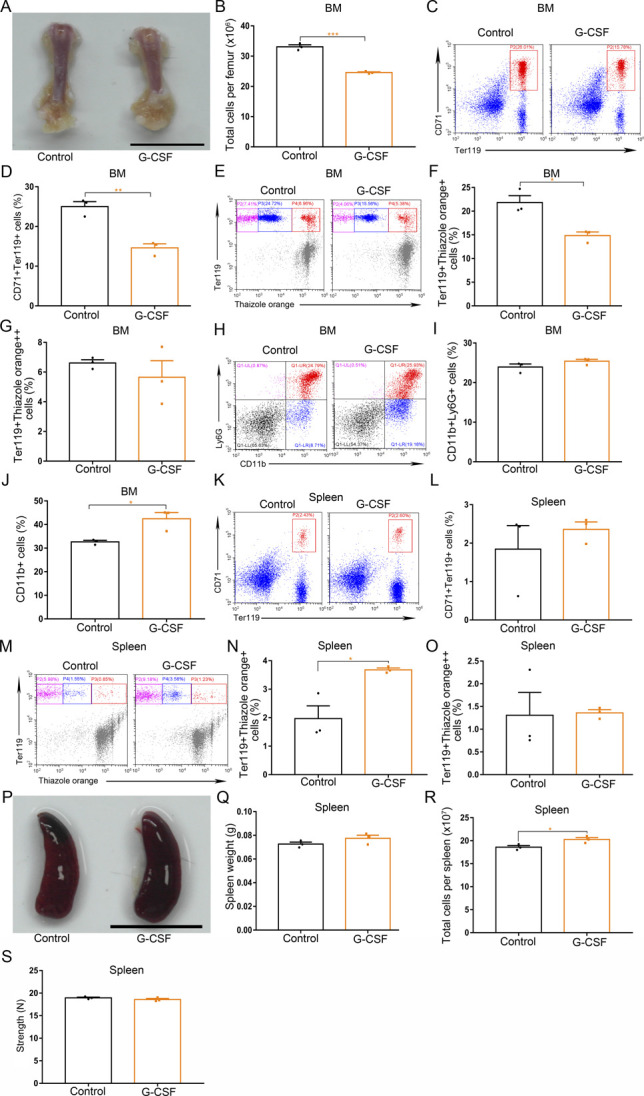
The effects of single dose of G-CSF (50 μg/kg) on BM and spleen. **(A)** Representative images of femurs from control and G-CSF–treated mice (scale bar = 1 cm). **(B)** Quantification of total BM cells. **(C)** Representative flow cytometric plots of CD71^+^Ter119^+^ cells in the BMs of control and G-CSF–treated mice. **(D)** Flow cytometric quantification of CD71^+^Ter119^+^ erythroid cells in the BMs of control and G-CSF–treated mice. **(E)** Representative flow cytometric profiles of Ter119-APC and thiazole orange–stained cells from the BMs of control and G-CSF–treated mice. **(F)** Flow cytometric quantification of reticulocytes (Ter119^+^ Thiazole orange^+^) in the BM of mice. **(G)** Flow cytometric quantification of erythroblasts (Ter119^+^ Thiazole orange^++^) in the BMs of mice. **(H)** Representative flow cytometric plots of CD11b^+^Ly6G^+^ cells in the BMs of control and G-CSF–treated mice. **(I)** Flow cytometric quantification of CD11b^+^Ly6G^+^ neutrophils in the BMs of control and G-CSF–treated mice. **(J)** Flow cytometric quantification of CD11b^+^ neutrophils in the BMs of control and G-CSF–treated mice. **(K)** Representative flow cytometric plots of CD71^+^Ter119^+^ cells in the spleens of control and G-CSF–treated mice. **(L)** Bar graphs showing quantification of CD71^+^Ter119^+^ cells in the spleens of control and G-CSF–treated mice. **(M)** Representative flow cytometric plots of Ter119-APC and thiazole orange–stained splenocytes from control and G-CSF–treated mice. **(N)** Flow cytometric quantification of reticulocytes (Ter119^+^ Thiazole orange^+^) in the spleens from different treatment groups. **(O)** Flow cytometric quantification of erythroblasts (Ter119^+^ Thiazole orange^++^) in the spleens from different treatment groups. **(P)** Macroscopic views of spleens in control and G-CSF–treated mice (scale bar = 1 cm). **(Q)** Average weights of spleens in control and G-CSF–treated mice. **(R)** Total cell numbers of spleens in control and G-CSF–treated mice. **(S)** Compressive strength of spleens from each group. n = 3/group (A, B, C, D, E, F, G, H, I, J, K, L, M, N, O, P, Q, R, S). *P < 0.05, **P < 0.01, ***P < 0.001.

Next, we evaluated erythropoiesis suppression by G-CSF in the bone marrow over time. Bone marrow erythropoiesis began to be significantly inhibited at 24 h after the first injection of G-CSF (50 μg/kg) and continued through G-CSF administration, as indicated by the pale bone color, decreased number of bone marrow cells, and decreased proportion of Ter119^+^CD71^+^ cells, reticulocytes, and erythroblasts (Fig 1L–R). We next explored whether depression of bone marrow erythropoiesis by G-CSF is reversible. Flow cytometric analyses showed that the Ter119^+^CD71^+^ cell, reticulocyte, and erythroblast populations in the bone marrow were restored after G-CSF withdrawal for 15 d and the bone color and bone marrow cell number also recovered after stopping treatment of mice with G-CSF (Fig S1A–G). We also treated mice with single dose of G-CSF (50 μg/kg), and we found that suppression of bone marrow erythropoiesis occurred in mice exposed to single dose of G-CSF (Fig S2A–G). Together, these observations suggest G-CSF reversibly suppresses erythropoiesis in the bone marrow in a dose- and time-dependent manner.

**Figure S3.**
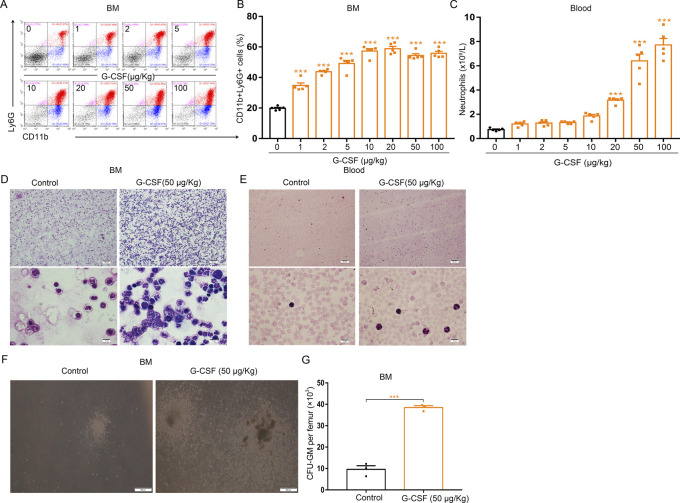
G-CSF treatment enhances granulopoiesis in the BM of mice. **(A, B)** Flow cytometric analysis of the BM neutrophils (CD11b^+^Ly6G^+^) in control and G-CSF–treated mice. **(A)** Representative flow cytometric plots of CD11b^+^Ly6G^+^ cells in the BMs of control and G-CSF–treated mice. **(B)** Flow cytometric quantification of CD11b^+^Ly6G^+^ neutrophils in the BMs of control and G-CSF–treated mice. **(C)** Peripheral neutrophil counts in control and G-CSF–treated mice. **(D)** Wright–Giemsa staining of BM cells from control and G-CSF–treated mice. Original magnification ×100 (upper panels); ×1,000 (lower panels) **(E)** Wright–Giemsa staining of peripheral blood cells from control and G-CSF–treated mice. **(F)** Representative fields of CFU-GM–derived colonies in BM cultures from control and G-CSF–treated mice (×100 magnification; scale bar = 200 μm). **(G)** Quantification of the number of CFU-GM–derived colonies in BM cultures from control and G-CSF treated mice. n = 5/group (A, B, C, D, E), n = 3/group (F, G).***P < 0.001.

G-CSF regulates neutrophil production and mobilization from the bone marrow. Therefore, we measured the neutrophil population in the bone marrow of G-CSF–treated mice using flow cytometry and found that the percentage of CD11b^+^Ly6G^+^ cells in the bone marrow gradually increased in response to increasing doses of G-CSF (Fig S3A and B). Analysis of hematological parameters showed G-CSF treatment led to more peripheral blood neutrophils in mice in a dose-dependent manner (Fig S3C). We also assessed blood and bone marrow smears after Wright–Giemsa staining by microscopy. Mice treated with G-CSF (50 μg/kg) had more neutrophils in the bone marrow and peripheral blood than the controls (Fig S3D and E). Quantification of CFU-GM showed that the numbers of CFU-GM derived from the bone marrow of G-CSF–treated mice were increased (Fig S3F and G). Administration of a single dose of G-CSF (50 μg/kg) led to an increase in the levels of CD11b^+^ cells, indicating that one dose of G-CSF initiates the granulopoiesis of bone marrow in mice (Fig S2H–J). These data suggest that G-CSF has different effects on erythropoiesis and granulopoiesis in the bone marrow of mice.

### G-CSF promotes splenic erythropoiesis of mice in a dose- and time-responsive fashion

**Figure 2.**
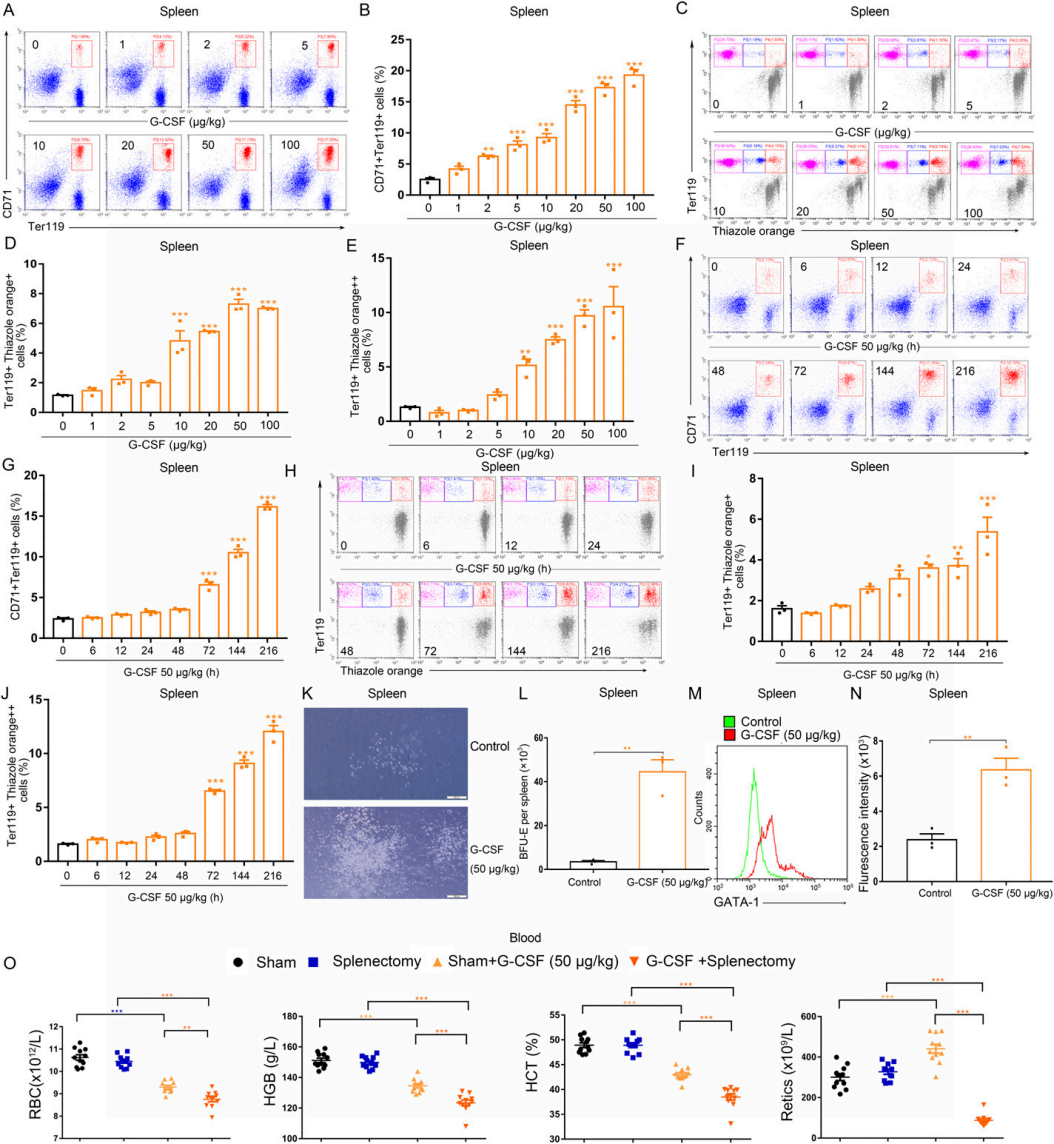
G-CSF enhances splenic erythropoiesis in a dose- and time-dependent manner. **(A)** Representative flow cytometric plots of CD71^+^Ter119^+^ cells in the spleens of control and G-CSF–treated mice. **(B)** Bar graphs showing quantification of CD71^+^Ter119^+^ cells in the spleens of control and G-CSF–treated mice. **(C)** Representative flow cytometric plots of Ter119-APC and thiazole orange-stained splenocytes from control and G-CSF–treated mice. **(D)** Flow cytometric quantification of reticulocytes (Ter119^+^ Thiazole orange^+^) in the spleens from different treatment groups. **(E)** Flow cytometric quantification of erythroblasts (Ter119^+^ Thiazole orange^++^) in the spleens from different treatment groups. **(F)** Representative flow cytometric plots of CD71^+^Ter119^+^ cells in the spleens of control and G-CSF–treated mice. **(G)** Bar graphs showing quantification of CD71^+^Ter119^+^ cells in the spleens of control and mice treated with G-CSF at different time points. **(H)** Representative flow cytometric profiles of splenocytes stained with anti-Ter119 and thiazole orange for each indicated group. **(I)** Flow cytometric quantification of reticulocytes (Ter119^+^ Thiazole orange^+^) in the spleens from different treatment groups. **(J)** Flow cytometric quantification of erythroblasts (Ter119^+^ Thiazole orange^++^) in the spleens from different treatment groups. **(K)** Representative fields of burst-forming unit-erythroid-derived colonies in cultures of splenocytes from control and G-CSF-treated mice (×100 magnification; scale bar = 200 μm). **(L)** Quantification of burst-forming unit erythroid-derived colonies in cultures of splenocytes from control and G-CSF–treated mice. **(M)** GATA-1 expression of splenocyte was measured by flow cytometry, and the representative histograms were shown. **(N)** Bar graphs showing mean fluorescence intensities. **(O)** Peripheral blood hematologic parameters of sham-operated mice, splenectomized mice, sham-operated mice treated with G-CSF, and splenectomized mice treated with G-CSF. Red blood cell (RBC), hemoglobin (HGB), hematocrit (HCT), and reticulocytes (Retics). n = 3/group (A, B, C, D, E, F, G, H, I, J, K, L, M, N), n = 10/group (O). *P < 0.05, **P < 0.01, ***P < 0.001 versus controls. Data are from one experiment representative of three experiments.

Splenic erythropoiesis can compensate for the repression of bone marrow erythropoiesis during stress (34). To assess the dose-responsive and time-dependent relationships between G-CSF and splenic erythropoiesis, we performed flow cytometric analysis using Ter119-APC and CD71-PE double staining as well as thiazole orange and Ter119-APC double staining. In sharp contrast to bone marrow, the spleen displayed a marked expansion of erythropoiesis in mice treated with G-CSF in a dose- and time-dependent manner, as indicated by the gradually increasing proportions of Ter119^+^CD71^+^ cells, reticulocytes, and erythroblasts (Fig 2A–J). The numbers of BFU-E colonies were also profoundly increased in the spleen of G-CSF-treated (50 μg/kg) mice (Fig 2K and L), confirming that G-CSF treatment promotes splenic erythropoiesis in mice. We also analyzed expression of GATA-1 in splenocytes by flow cytometry and found an increase in mice treated with G-CSF (50 μg/kg) (Fig 2M and N). This expansion of splenic erythropoiesis was maintained throughout G-CSF administration and gradually contracted following recovery of bone marrow erythropoiesis after terminating G-CSF administration (Fig S1H–L). Single-dose administration of G-CSF (50 μg/kg) was not able to trigger the splenic erythropoiesis (Fig S2K–O). To further confirm the crucial role of splenic erythropoiesis in mice treated with G-CSF, we performed splenectomies in mice, treated the mice with G-CSF (50 μg/kg) for 15 d, and then analyzed the hematologic parameters in peripheral blood. The G-CSF–treated mice that underwent a sham operation developed mild anemia with low red blood cell counts, hemoglobin concentrations, and hematocrits, and increased reticulocyte counts. G-CSF treatment significantly reduced the red blood cell counts, hemoglobin concentrations, hematocrits, and reticulocyte counts in mice that had undergone splenectomies (Fig 2O), indicating that splenic erythropoiesis is indispensable for maintaining red blood cell homeostasis in G-CSF–treated mice.

### Systemic inflammation up-regulates G-CSF levels, inhibits bone marrow erythropoiesis, and promotes splenic erythropoiesis in mice

**Figure 3.**
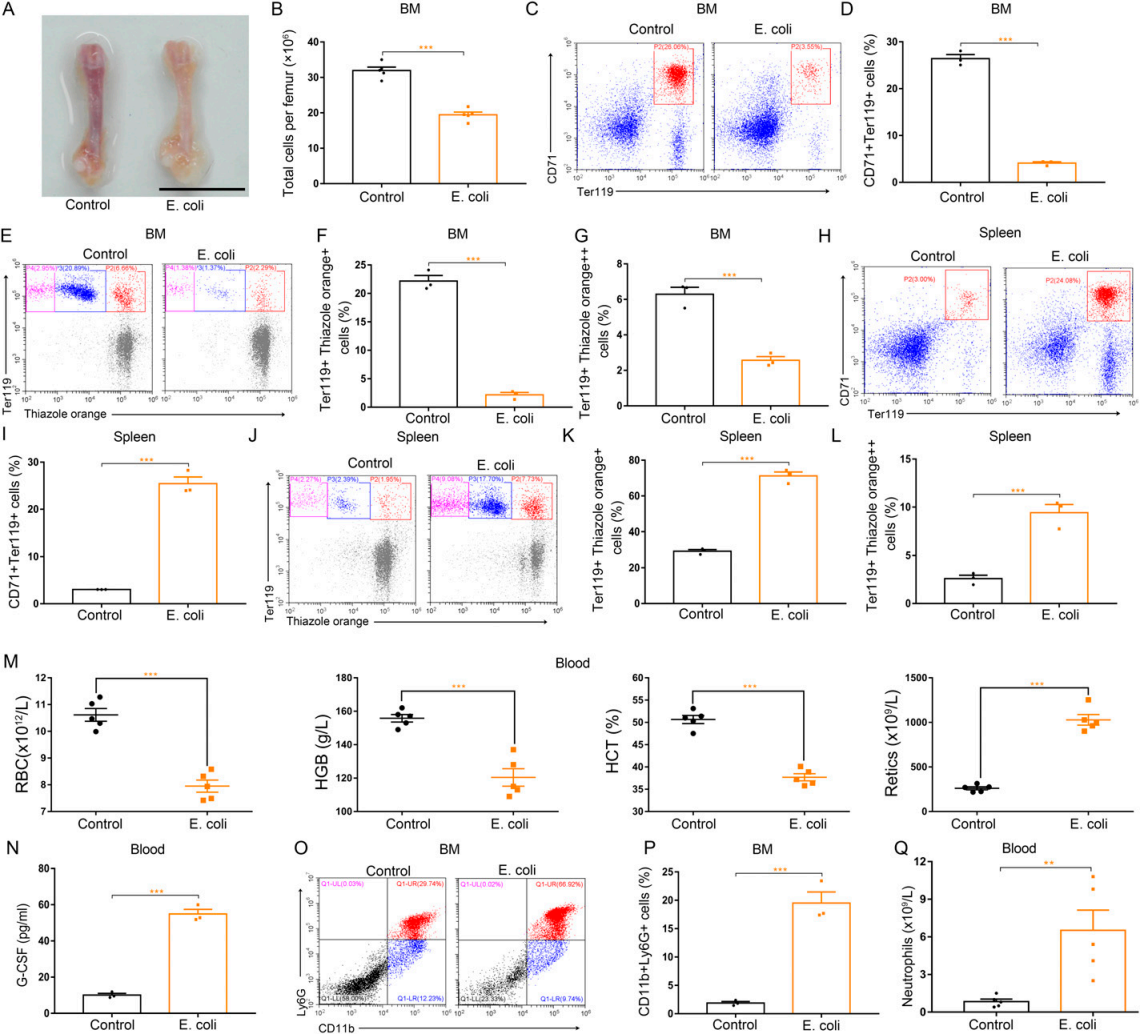
Infection of mice with E. coli impairs BM erythropoiesis, enhances splenic erythropoiesis, and promotes BM granulopoiesis. **(A)** Representative images of femurs from control mice and *E. coli*–infected mice (scale bar = 1 cm). **(B)** Quantification of total BM cells. **(C)** Representative flow cytometric plots of CD71^+^Ter119^+^ cells in the BMs of control mice and *E. coli*–infected mice. **(D)** Flow cytometric quantification of CD71^+^Ter119^+^ erythroid cells in the BMs of control and *E. coli*-infected mice. **(E)** Representative flow cytometric profiles of Ter119-APC and thiazole orange-stained cells from the BM of control mice and *E. coli*–infected mice. **(F)** Flow cytometric quantification of reticulocytes (Ter119^+^Thiazole orange^+^) in the BM of mice. **(G)** Flow cytometric quantification of erythroblasts (Ter119^+^ Thiazole orange^++^) in the BMs of mice. **(H)** Representative flow cytometric plots of CD71^+^Ter119^+^ cells in the spleens of control and *E. coli*-infected mice. **(I)** Bar graphs showing quantification of CD71^+^Ter119^+^ cells in the spleens of control and *E. coli*–infected mice. **(J)** Representative flow cytometric plots of Ter119-APC and thiazole orange–stained splenocytes from control and *E. coli*–infected mice. **(K)** Flow cytometric quantification of reticulocytes (Ter119^+^ Thiazole orange^+^) in the spleens from control and *E. coli*–infected mice. **(L)** Flow cytometric quantification of erythroblasts (Ter119^+^ Thiazole orange^++^) in the spleens from control and *E. coli*-infected mice. **(M)** Peripheral blood hematologic parameters of control mice and *E. coli*-infected mice. Red blood cell (RBC), hemoglobin (HGB), hematocrit (HCT), and reticulocytes (Retics). **(N)** Serum concentrations of G-CSF in control and *E. coli*–infected mice. **(O)** Representative flow cytometric plots of CD11b^+^Ly6G^+^ cells in the BMs of control and *E. coli*-infected mice. **(P)** Flow cytometric quantification of CD11b^+^Ly6G^+^ neutrophils in the BMs of control and *E. coli*–infected mice. **(Q)** Peripheral neutrophil counts in control and *E. coli*–infected mice. n = 5/group (A, B, M, Q), n = 3/group (C, D, E, F, G, H, I, J, K, L, N, O, P). **P < 0.01, ***P < 0.001.

**Figure S4.**
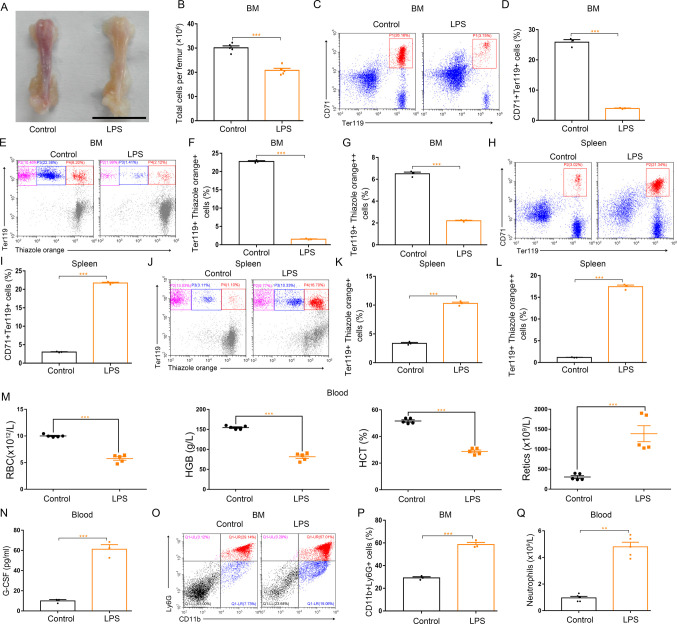
Treatment of mice with LPS impairs BM erythropoiesis, enhances splenic erythropoiesis, and promotes BM granulopoiesis. **(A)** Representative images of femurs from control and LPS-treated mice (scale bar = 1 cm). **(B)** Quantification of total BM cells. **(C)** Representative flow cytometric plots of CD71^+^Ter119^+^ cells in the BMs of control and LPS-treated mice. **(D)** Flow cytometric quantification of CD71^+^Ter119^+^ erythroid cells in the BMs of control and LPS-treated mice. **(E)** Representative flow cytometric profiles of Ter119-APC and thiazole orange-stained cells from the BM of control and LPS-treated mice. **(F)** Flow cytometric quantification of reticulocytes (Ter119^+^ Thiazole orange^+^) in the BM of mice. **(G)** Flow cytometric quantification of erythroblasts (Ter119^+^ Thiazole orange^++^) in the BMs of mice. **(H)** Representative flow cytometric plots of CD71^+^Ter119^+^ cells in the spleens of control and LPS-treated mice. **(I)** Bar graphs showing quantification of CD71^+^Ter119^+^ cells in the spleens of control and LPS-treated mice. **(J)** Representative flow cytometric profiles of Ter119-APC and thiazole orange-stained cells from the spleen of control and LPS-treated mice. **(K)** Flow cytometric quantification of reticulocytes (Ter119^+^ Thiazole orange^+^) in the spleens from control and LPS-treated mice. **(L)** Flow cytometric quantification of erythroblasts (Ter119^+^ Thiazole orange^++^) in the spleens from control and LPS-treated mice. **(M)** Peripheral blood hematologic parameters of control and LPS-treated mice. Red blood cell (RBC), hemoglobin (HGB), hematocrit (HCT), and reticulocytes (Retics). **(N)** Serum concentrations of G-CSF in control and LPS-treated mice. **(O)** Representative flow cytometric plots of CD11b^+^Ly6G^+^ cells in the BMs of control and LPS-treated mice. **(P)** Flow cytometric quantification of CD11b^+^Ly6G^+^ neutrophils in the BMs of control and LPS-treated mice. **(Q)** Peripheral neutrophil counts in control and LPS-treated mice. n = 5/group (A, B, M, Q), n = 3/group (C, D, E, F, G, H, I, J, K, L, N, O, P). **P < 0.01, ***P < 0.001.

**Figure S5.**
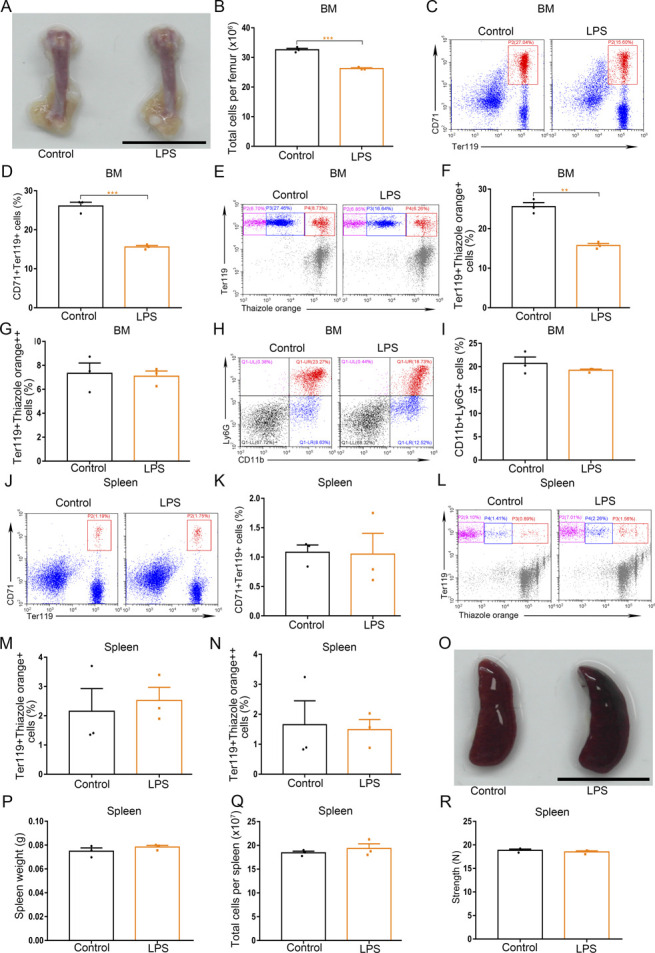
The effects of single treatment of LPS (0.5 mg/kg) on BM and spleen. **(A)** Representative images of femurs from control and LPS-treated mice (scale bar = 1 cm). **(B)** Quantification of total BM cells. **(C)** Representative flow cytometric plots of CD71^+^Ter119^+^ cells in the BMs of control and LPS-treated mice. **(D)** Flow cytometric quantification of CD71^+^Ter119^+^ erythroid cells in the BMs of control and LPS-treated mice. **(E)** Representative flow cytometric profiles of Ter119-APC and thiazole orange-stained cells from the BMs of control and LPS-treated mice. **(F)** Flow cytometric quantification of reticulocytes (Ter119^+^ Thiazole orange^+^) in the BM of mice. **(G)** Flow cytometric quantification of erythroblasts (Ter119^+^ Thiazole orange^++^) in the BMs of mice. **(H)** Representative flow cytometric plots of CD11b^+^Ly6G^+^ cells in the BMs of control and LPS-treated mice. **(I)** Flow cytometric quantification of CD11b^+^Ly6G^+^ neutrophils in the BMs of control and LPS-treated mice. **(J)** Representative flow cytometric plots of CD71^+^Ter119^+^ cells in the spleens of control and LPS-treated mice. **(K)** Bar graphs showing quantification of CD71^+^Ter119^+^ cells in the spleens of control and LPS-treated mice. **(L)** Representative flow cytometric plots of Ter119-APC and thiazole orange-stained splenocytes from control and LPS-treated mice. **(M)** Flow cytometric quantification of reticulocytes (Ter119^+^ Thiazole orange^+^) in the spleens from different treatment groups. **(N)** Flow cytometric quantification of erythroblasts (Ter119^+^ Thiazole orange^++^) in the spleens from different treatment groups. **(O)** Macroscopic views of spleens in control and LPS-treated mice (scale bar = 1 cm). **(P)** Average weights of spleens in control and LPS-treated mice. **(Q)** Total cell numbers of spleens in control and LPS-treated mice. **(R)** Compressive strength of spleens from each group. n = 3/group (A, B, C, D, E, F, G, H, I, J, K, L, M, N, O, P, Q, R). **P < 0.01, ***P < 0.001.

**Figure S6.**
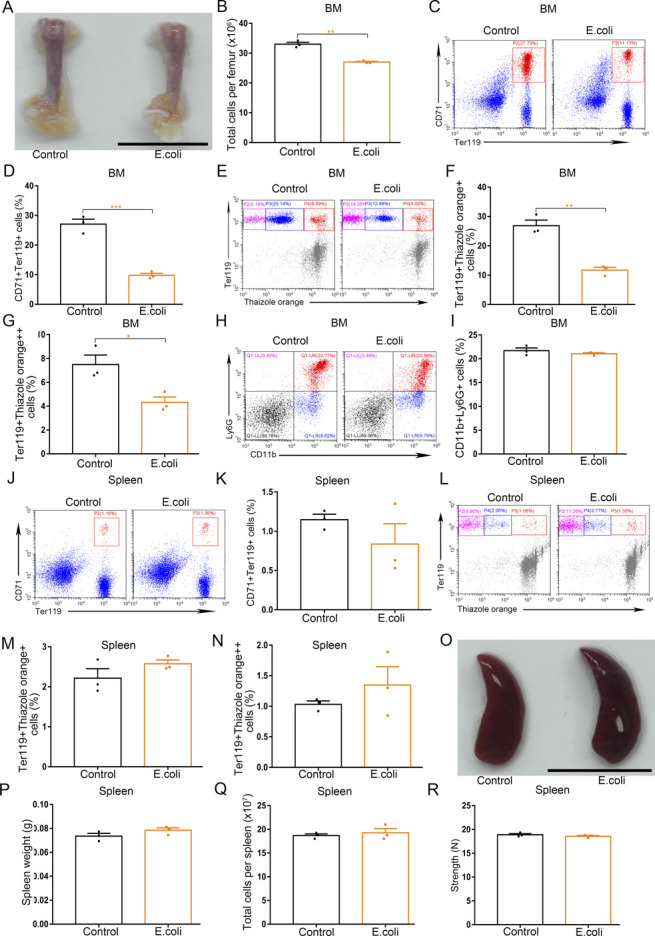
The effects of single infection of *E. coli* (1 × 106) on BM and spleen. **(A)** Representative images of femurs from control and *E. coli*–infected mice (scale bar = 1 cm). **(B)** Quantification of total BM cells. **(C)** Representative flow cytometric plots of CD71^+^Ter119^+^ cells in the BMs of control and *E. coli*–infected mice. **(D)** Flow cytometric quantification of CD71^+^Ter119^+^ erythroid cells in the BMs of control and *E. coli*–infected mice. **(E)** Representative flow cytometric profiles of Ter119-APC and thiazole orange–stained cells from the BMs of control and *E. coli*–infected mice. **(F)** Flow cytometric quantification of reticulocytes (Ter119^+^ Thiazole orange^+^) in the BM of mice. **(G)** Flow cytometric quantification of erythroblasts (Ter119^+^ Thiazole orange^++^) in the BMs of mice. **(H)** Representative flow cytometric plots of CD11b^+^Ly6G^+^ cells in the BMs of control and *E. coli*-infected mice. **(I)** Flow cytometric quantification of CD11b^+^Ly6G^+^ neutrophils in the BMs of control and *E. coli*–infected mice. **(J)** Representative flow cytometric plots of CD71^+^Ter119^+^ cells in the spleens of control and *E. coli*–infected mice. **(K)** Bar graphs showing quantification of CD71^+^Ter119^+^ cells in the spleens of control and *E. coli*–infected mice. **(L)** Representative flow cytometric plots of Ter119-APC and thiazole orange–stained splenocytes from control and *E. coli*–infected mice. **(M)** Flow cytometric quantification of reticulocytes (Ter119^+^ Thiazole orange^+^) in the spleens from different treatment groups. **(N)** Flow cytometric quantification of erythroblasts (Ter119^+^ Thiazole orange^++^) in the spleens from different treatment groups. **(O)** Macroscopic views of spleens in control and *E. coli*–infected mice (scale bar = 1 cm). **(P)** Average weights of spleens in control and *E. coli*–infected mice. **(Q)** Total cell numbers of spleens in control and *E. coli*-infected mice. **(R)** Compressive strength of spleens from each group. n = 3/group (A, B, C, D, E, F, G, H, I, J, K, L, M, N, O, P, Q, R). *P < 0.05, **P < 0.01, ***P < 0.001.

To examine inflammation-driven dysfunction of erythropoiesis, we used *Escherichia coli* (*E. coli*) and LPS to induce systemic inflammation separately. Both systemic infection of mice with *E. coli* and treatment of mice with LPS resulted in regression of bone marrow erythropoiesis and enhancement of splenic erythropoiesis (Figs 3A–L and S4A–L). The *E. coli*–infected mice and LPS-treated mice were both phenotypically anemic with low red blood cell counts, hemoglobin concentrations, and hematocrits, and high-reticulocyte counts (Figs 3M and S4M). Accumulating evidence shows G-CSF shapes host immunity by enhancing the production and function of neutrophils in response to infection-related inflammatory stress. Therefore, we determined whether G-CSF is associated with an alteration in erythropoiesis in mice during inflammatory processes. For this purpose, the serum G-CSF levels were analyzed in mice infected with *E. coli* or treated with LPS. Both systemic *E. coli* infection and LPS treatment increased serum levels of G-CSF (Figs 3N and S4N). We also measured the populations of neutrophils in the bone marrow and peripheral blood of *E. coli*–infected and LPS-treated mice and found the numbers of neutrophils (CD11b^+^Ly6G^+^) in both the bone marrow and peripheral blood increased in response to *E. coli* infection and LPS stimulation (Figs 3O–Q and S4O–Q), indicating that LPS stimulates the secretion of G-CSF and initiates emergency granulopoiesis in bone marrow during systemic inflammation. Single treatment of LPS and single infection of *E. coli* were associated with regression of bone marrow erythropoiesis, but did not trigger emergency granulopoiesis of bone marrow and splenic erythropoiesis (Figs S5A–N and S6A–N).

### G-CSF neutralization reverses the functional changes of erythropoiesis in the bone marrow and spleens of LPS-treated mice

**Figure 4.**
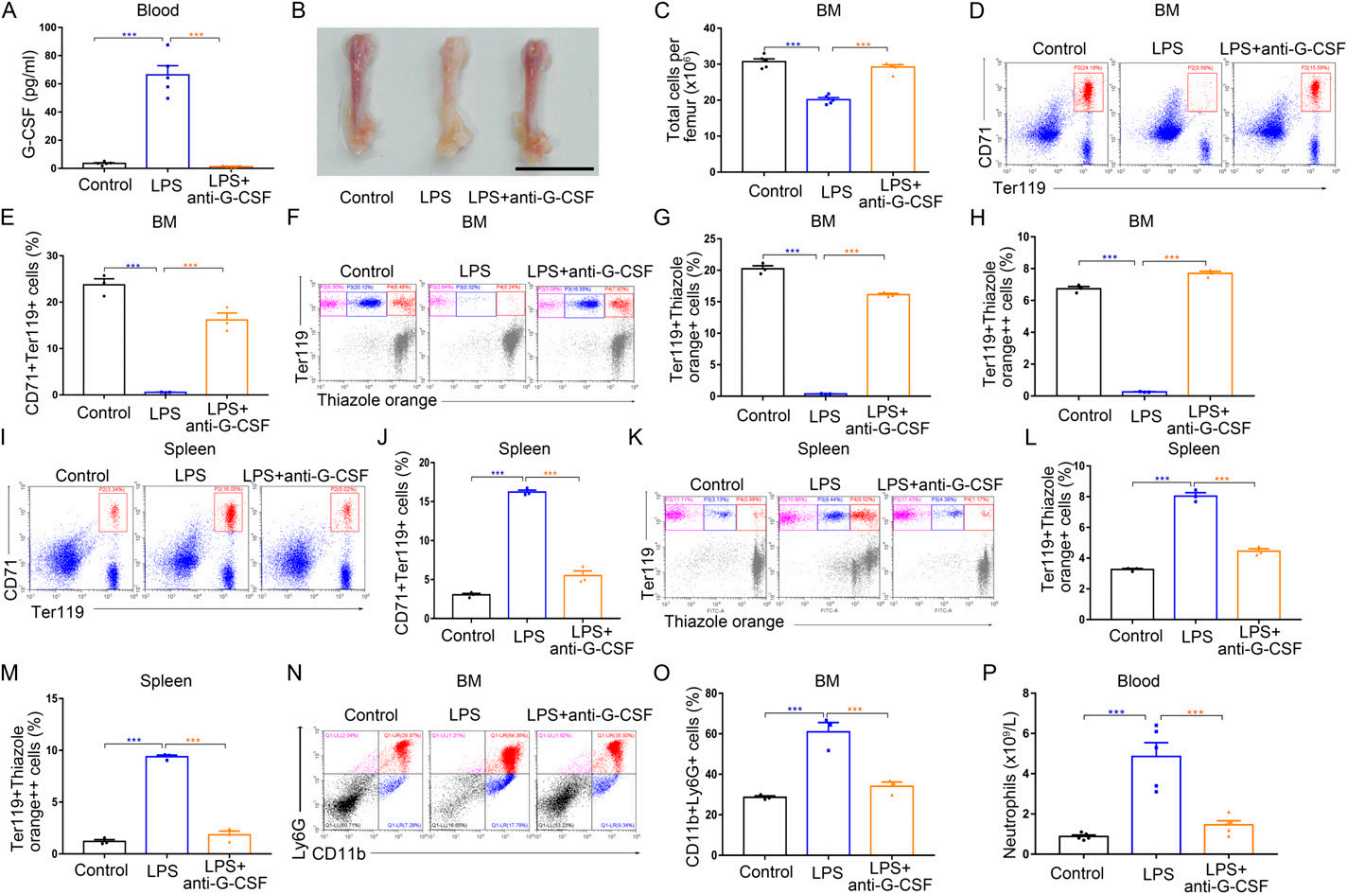
G-CSF neutralization decreases BM granulopoiesis, restores BM erythropoiesis, and suppresses splenic erythropoiesis in LPS-treated mice. **(A)** Serum concentrations of G-CSF in control mice, LPS-treated mice, and LPS-treated mice with anti-G-CSF treatment. **(B)** Representative images of femurs from control mice, LPS-treated mice, and LPS-treated mice with anti-G-CSF treatment (scale bar = 1 cm). **(C)** Quantification of total BM cells. **(D)** Representative flow cytometric plots of CD71^+^Ter119^+^ cells in the BMs of control mice, LPS-treated mice, and LPS-treated mice with anti-G-CSF treatment. **(E)** Flow cytometric quantification of CD71^+^Ter119^+^ erythroid cells in the BMs of control mice, LPS-treated mice, and LPS-treated mice with anti–G-CSF treatment. **(F)** Representative flow cytometric profiles of Ter119-APC and thiazole orange–stained cells from the BM of control mice, LPS-treated mice, and LPS-treated mice with anti–G-CSF treatment. **(G)** Flow cytometric quantification of reticulocytes (Ter119^+^ Thiazole orange^+^) in the BM of mice. **(H)** Flow cytometric quantification of erythroblasts (Ter119^+^ Thiazole orange^++^) in the BMs of mice. **(I)** Representative flow cytometric plots of CD71^+^Ter119^+^ cells in the spleens of control mice, LPS-treated mice and LPS-treated mice with anti-G-CSF treatment. **(J)** Bar graphs showing quantification of CD71^+^Ter119^+^ cells in the spleens of control mice, LPS-treated mice, and LPS-treated mice with anti–G-CSF treatment. **(K)** Representative flow cytometric profiles of Ter119-APC and thiazole orange–stained cells from the spleen of control mice, LPS-treated mice, and LPS-treated mice with anti–G-CSF treatment. **(L)** Flow cytometric quantification of reticulocytes (Ter119^+^ Thiazole orange^+^) in the spleens from different treatment groups. **(M)** Flow cytometric quantification of erythroblasts (Ter119^+^ Thiazole orange^++^) in the spleens from different treatment groups. **(N)** Representative flow cytometric plots of CD11b^+^Ly6G^+^ cells in the BMs of control mice, LPS-treated mice, and LPS-treated mice with anti–G-CSF treatment. **(O)** Flow cytometric quantification of CD11b^+^Ly6G^+^ neutrophils in the BMs of control mice, LPS-treated mice, and LPS-treated mice with anti–G-CSF treatment. **(P)** Peripheral neutrophil counts in control mice, LPS-treated mice, and LPS-treated mice with anti–G-CSF treatment. n = 3/group (D, E, F, G, H, I, J, K, L, M, N, O), n = 5/group (A, B, C, P). ***P < 0.001.

**Figure S7.**
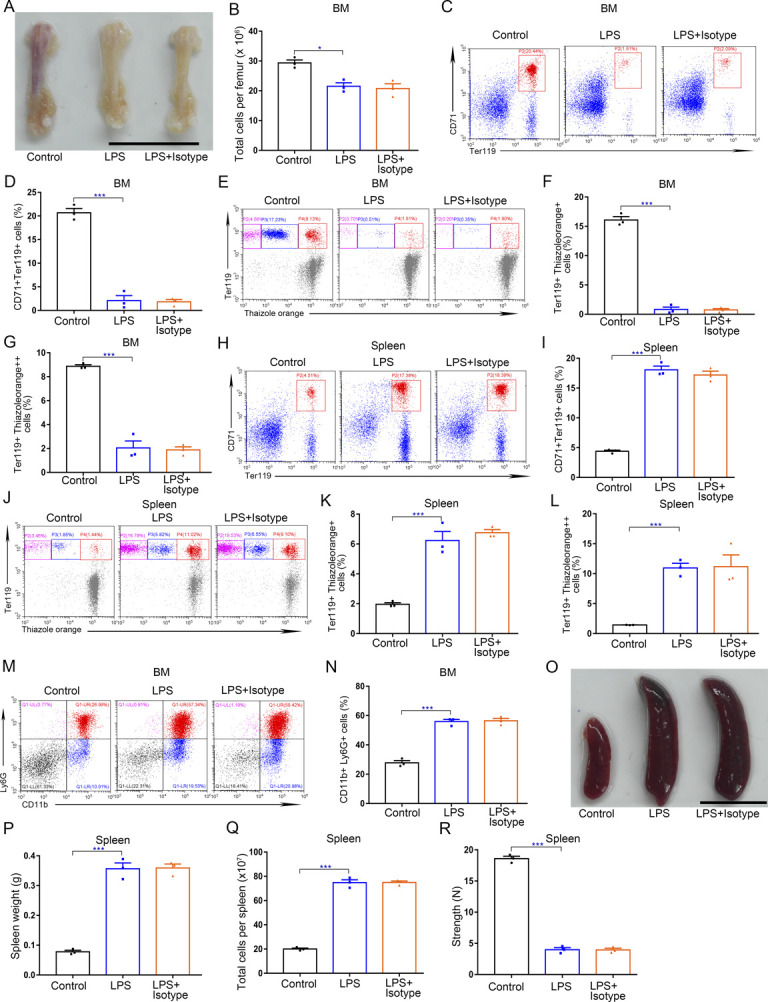
Anti–G-CSF isotype control has no influence on BM and spleen that changed by LPS treatment. **(A)** Representative images of femurs from WT control mice, LPS-treated mice and LPS-treated mice with isotype treatment (scale bar = 1 cm). **(B)** Quantification of total BM cells. **(C)** Representative flow cytometric plots of CD71^+^Ter119^+^ cells in the BMs of control, LPS-treated mice, and LPS-treated mice with isotype treatment. **(D)** Flow cytometric quantification of CD71^+^Ter119^+^ erythroid cells in the BMs of control, LPS-treated mice, and LPS-treated mice with isotype treatment. **(E)** Representative flow cytometric profiles of Ter119-APC and thiazole orange-stained cells from the BMs of control, LPS-treated mice, and LPS-treated mice with isotype treatment. **(F)** Flow cytometric quantification of reticulocytes (Ter119^+^ Thiazole orange^+^) in the BM of mice. **(G)** Flow cytometric quantification of erythroblasts (Ter119^+^ Thiazole orange^++^) in the BMs of mice. **(H)** Representative flow cytometric plots of CD71^+^Ter119^+^ cells in the spleens of control, LPS-treated mice, and LPS-treated mice with isotype treatment. **(I)** Flow cytometric quantification of CD71^+^Ter119^+^ erythroid cells in the spleens of control, LPS-treated mice, and LPS-treated mice with isotype treatment. **(J)** Representative flow cytometric profiles of Ter119-APC and thiazole orange–stained cells from the spleens of control, LPS-treated mice, and LPS-treated mice with isotype treatment. **(K)** Flow cytometric quantification of reticulocytes (Ter119^+^ Thiazole orange^+^) in the spleens of mice. **(L)** Flow cytometric quantification of erythroblasts (Ter119^+^ Thiazole orange^++^) in the spleens of mice. **(M)** Representative flow cytometric plots of CD11b^+^Ly6G^+^ cells in the BMs of control, LPS-treated mice, and LPS-treated mice with isotype treatment. **(N)** Flow cytometric quantification of CD11b^+^Ly6G^+^ cells in the BMs of mice. **(O)** Representative images of spleens from WT control mice, LPS-treated mice, and LPS-treated mice with isotype treatment. **(P)** Average weights of spleens from each group. **(Q)** Total cell numbers of spleens from each group. **(R)** Compressive strength of spleens from each group. n = 3/group (A, B, C, D, E, F, G, H, I, J, K, L, M, N, O, P, Q, R). *P < 0.05, ***P < 0.001.

We then hypothesized that G-CSF impairs bone marrow erythropoiesis and promotes splenic erythropoiesis in mice during LPS-induced inflammation. To test this hypothesis, we examined erythropoiesis in the bone marrow and spleens in the setting of antibody-mediated neutralization of G-CSF. Anti–G-CSF treatment reduced serum G-CSF concentrations in LPS-treated mice (Fig 4A), restored erythropoiesis in the bone marrow (Figs 4B–H and S7A–G), and reversed LPS-induced splenic erythropoiesis (Figs 4I–M and S7H–L), indicating that systemic inflammation influences erythropoiesis in the body by increasing G-CSF production. As expected, G-CSF neutralization blocked emergency granulopoiesis in the bone marrow (Figs 4N and O and S7M and N) and decreased blood neutrophil counts (Fig 4P).

### Knockout of *TLR4* down-regulates G-CSF levels and restores the balance of erythropoiesis in LPS-treated mice

**Figure 5.**
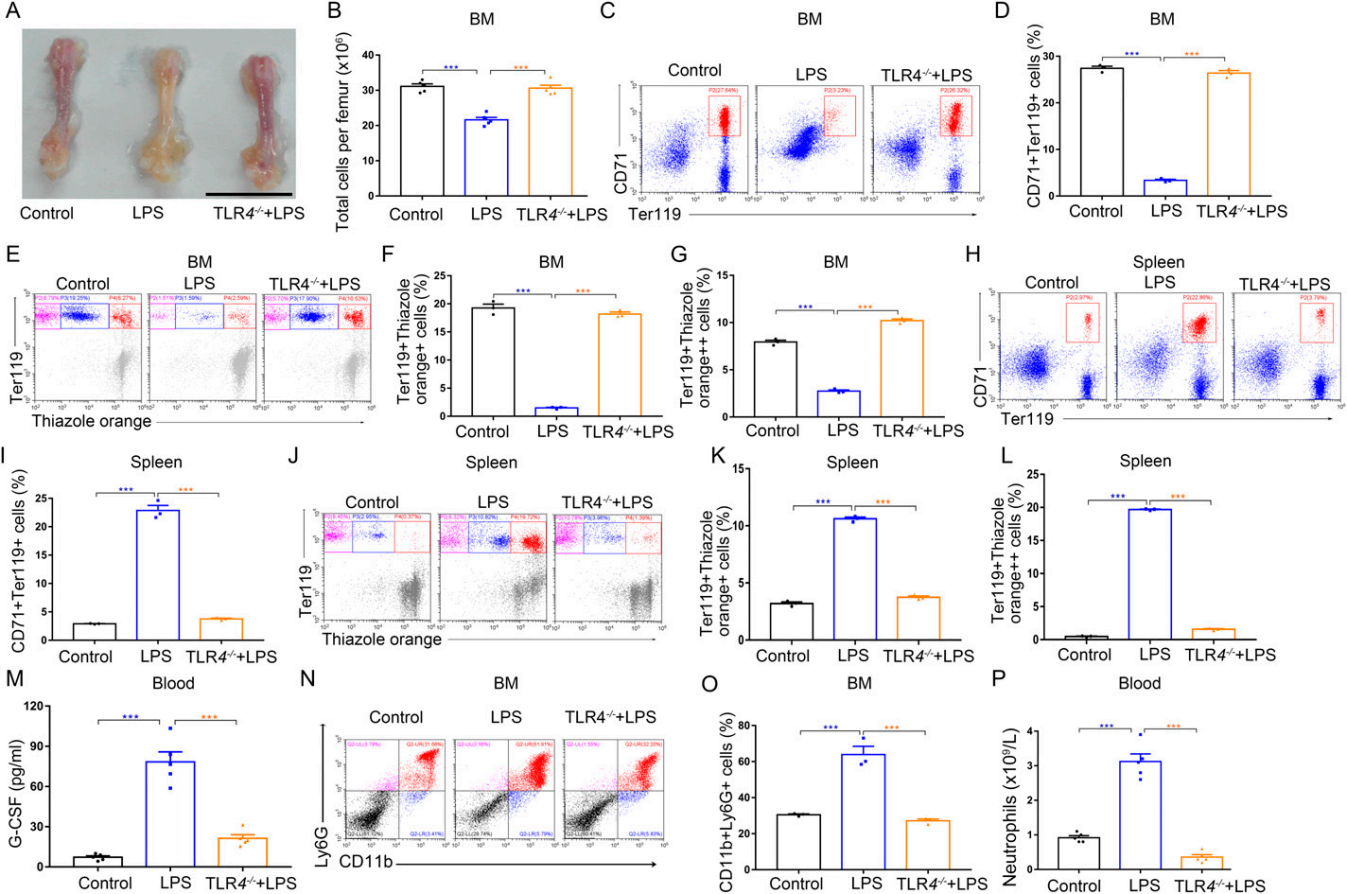
*TLR4* knockout down-regulates G-CSF expression, abolishes the suppression of BM erythropoiesis, inhibits splenic erythropoiesis, and blocks emergency granulopoiesis in LPS-treated mice. **(A)** Representative images of femurs from WT control mice, LPS-treated mice, and *TLR4*^*−/−*^ mice treated with LPS (scale bar = 1 cm). **(B)** Quantification of total BM cells. **(C)** Representative flow cytometric plots of CD71^+^Ter119^+^ cells in the BMs of WT control mice, LPS-treated mice, and *TLR4*^*−/−*^ mice treated with LPS. **(D)** Flow cytometric quantification of CD71^+^Ter119^+^ erythroid cells in the BMs of WT control mice, LPS-treated mice, and *TLR4*^*−/−*^ mice treated with LPS. **(E)** Representative flow cytometric profiles of Ter119-APC and thiazole orange–stained cells from the BM of WT control mice, LPS-treated mice, and *TLR4*^*−/−*^ mice treated with LPS. **(F)** Flow cytometric quantification of reticulocytes (Ter119^+^ Thiazole orange^+^) in the BM of mice. **(G)** Flow cytometric quantification of erythroblasts (Ter119^+^ Thiazole orange^++^) in the BMs of mice. **(H)** Representative flow cytometric plots of CD71^+^Ter119^+^ cells in the spleens of WT control mice, LPS-treated mice, and *TLR4*^*−/−*^ mice treated with LPS. **(I)** Bar graphs showing quantification of CD71^+^Ter119^+^ cells in the spleens of WT control mice, LPS-treated mice and *TLR4*^*−/−*^ mice treated with LPS. **(J)** Representative flow cytometric profiles of Ter119-APC and thiazole orange-stained cells from the spleen of WT control mice, LPS-treated mice, and *TLR4*^*−/−*^ mice treated with LPS. **(K)** Flow cytometric quantification of reticulocytes (Ter119^+^ Thiazole orange^+^) in the spleens from different treatment groups. **(L)** Flow cytometric quantification of erythroblasts (Ter119^+^ Thiazole orange^++^) in the spleens from WT control mice, LPS-treated mice, and *TLR4*^*−/−*^ mice treated with LPS. **(M)** Serum concentrations of G-CSF in WT control mice, LPS-treated mice, and *TLR4*^*−/−*^ mice treated with LPS. **(N)** Representative flow cytometric plots of CD11b^+^Ly6G^+^ cells in the BMs of WT control mice, LPS-treated mice, and *TLR4*^*−/−*^ mice treated with LPS. **(O)** Flow cytometric quantification of CD11b^+^Ly6G^+^ neutrophils in the BMs of WT control mice, LPS-treated mice, and *TLR4*^*−/−*^ mice treated with LPS. **(P)** Peripheral neutrophil counts in WT control mice, LPS-treated mice, and *TLR4*^*−/−*^ mice treated with LPS. n = 5/group (A, B, M, P), n = 3/group (C, D, E, F, G, H, I, J, K, L, N, O). ***P < 0.001.

TLRs, which are expressed in immune effector cells and endothelial cells, trigger inflammatory responses by inducing production of inflammatory mediators. Endothelial cells can translate the signal of systemically spread pathogens into G-CSF release via TLR4 signaling after LPS challenge and are the prime sources of G-CSF that drives emergency bone marrow granulopoiesis (4, 35, 36). We speculated that TLR4 is the upstream factor that initiates the LPS-induced alteration of erythropoiesis. To test this hypothesis, we investigated erythropoiesis in the bone marrow and spleen during LPS treatment in *TLR4*-knockout mice. We observed that TLR4 deficiency almost prevented repression of bone marrow erythropoiesis (Fig 5A–G) and blocked splenic erythropoiesis in LPS-treated mice (Fig 5H–L). Next, we investigated the potential impact of TLR4 on G-CSF production and granulopoiesis of bone marrow in LPS-treated mice. In line with previous reports, *TLR4*^−/−^ mice showed low serum levels of G-CSF (Fig 5M), impaired emergency granulopoiesis of bone marrow (Fig 5N and O), and a dramatic decrease in blood neutrophil counts after LPS treatment (Fig 5P). Together, our findings suggest that TLR4-mediated G-CSF production plays an essential role in inflammation-associated splenic erythropoiesis.

### G-CSF–induced splenic erythropoiesis relies on high levels of EPO

**Figure 6.**
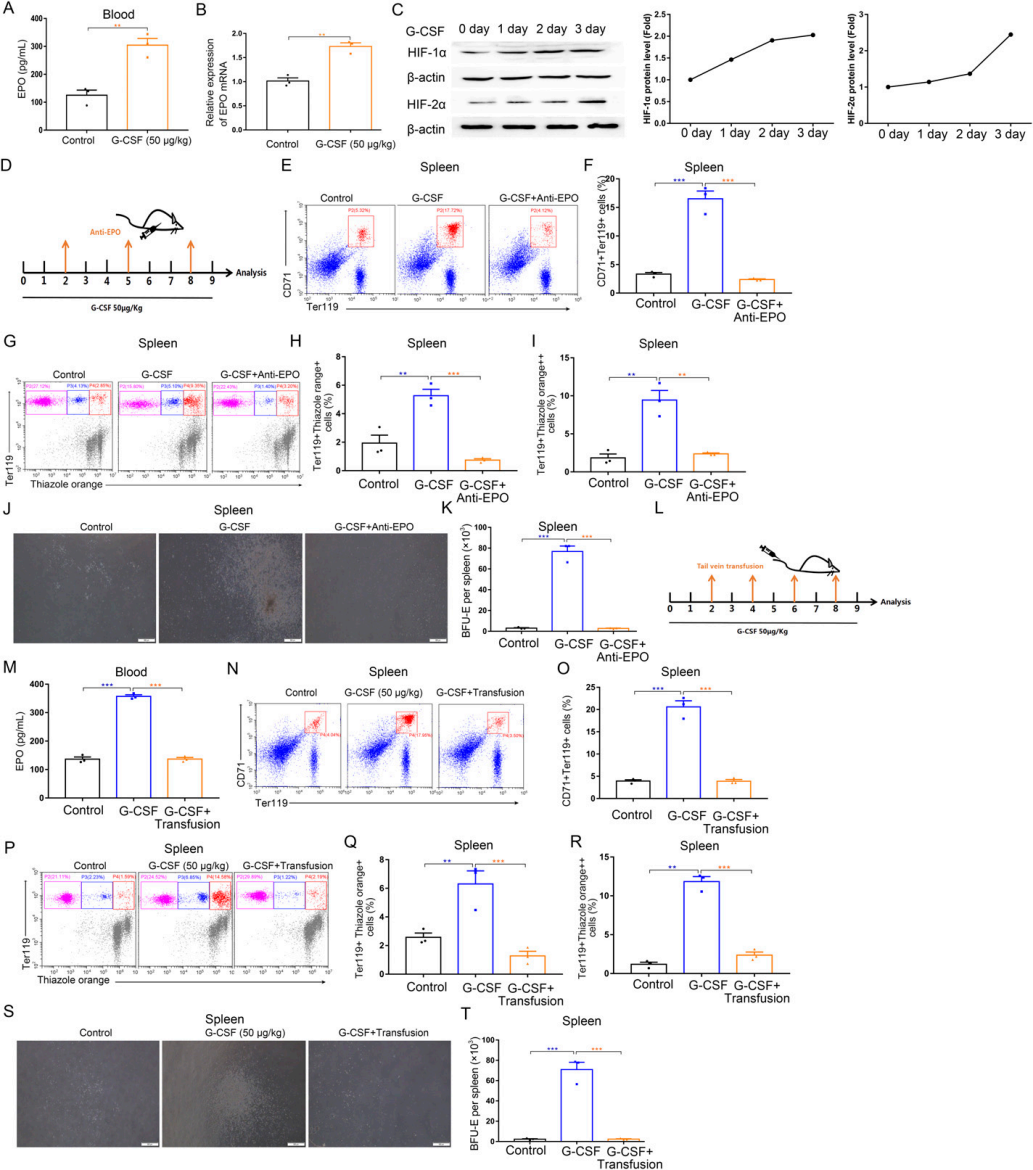
EPO mediates G-CSF–induced splenic erythropoiesis. **(A)** Serum levels of EPO in control and G-CSF–treated mice. The mice were treated with G-CSF for 3 d. **(B)** Quantification of kidney EPO mRNA by quantitative reverse-transcribed polymerase chain reaction, normalized to β-actin mRNA. The mice were treated with G-CSF for 3 d. **(C)** Western blot analyses for the levels of HIF-1α and HIF-2α in kidneys. The mice were treated with G-CSF for 0, 1, 2, 3 d. Protein lysates were prepared from the kidney, and Western blot analyses were performed. **(D)** Protocol of anti-EPO blocking antibody treatment in G-CSF-treated mice. **(E, F, G, H, I)** Flow cytometric analysis of the splenic erythropoiesis in control mice, G-CSF–treated mice, and G-CSF–treated mice with anti-EPO treatment. **(E)** Representative flow cytometric plots of CD71^+^Ter119^+^ cells in the spleens. **(F)** Bar graphs showing quantification of CD71^+^Ter119^+^ cells in the spleens from different treatment groups. **(G)** Representative flow cytometric plots of Ter119-APC and thiazole orange-stained splenocytes from control mice, G-CSF–treated mice, and G-CSF–treated mice with anti-EPO treatment. **(H)** Flow cytometric quantification of reticulocytes (Ter119^+^ Thiazole orange^+^) in the spleens from different treatment groups. **(I)** Flow cytometric quantification of erythroblasts (Ter119^+^ Thiazole orange^++^) in the spleens from different treatment groups. **(J)** Representative fields of burst-forming unit erythroid (BFU-E)–derived colonies in spleen cultures from control, G-CSF–treated mice, and G-CSF–treated mice with anti-EPO (×100 magnification; scale bar = 200 μm). **(K)** Quantification of the number of BFU-E-derived colonies in spleen cultures from different groups. **(L)** Protocol of red blood cell transfusions in G-CSF–treated mice. **(M)** Serum levels of EPO in WT control mice, G-CSF–treated mice, and G-CSF–treated mice with red blood cell transfusion. **(N, O, P, Q, R)** Flow cytometric analysis of the splenic erythropoiesis in control mice, G-CSF–treated mice, and G-CSF–treated mice with red blood cell transfusion. **(N)** Representative flow cytometric plots of CD71^+^Ter119^+^ cells in the spleens. **(O)** Bar graphs showing quantification of CD71^+^Ter119^+^ cells in the spleens from different treatment groups. **(P)** Representative flow cytometric profiles of splenocytes stained with anti-Ter119 and thiazole orange for each indicated group. **(Q)** Flow cytometric quantification of reticulocytes (Ter119^+^ Thiazole orange^+^) in the spleens from different treatment groups. **(R)** Flow cytometric quantification of erythroblasts (Ter119^+^ Thiazole orange^++^) in the spleens from different treatment groups. **(S)** Representative fields of BFU-E–derived colonies in spleen cultures from control, G-CSF–treated mice, and G-CSF–treated mice with transfusion (×100 magnification; scale bar = 200 μm). **(T)** Quantification of the number of BFU-E–derived colonies in spleen cultures from different groups. n = 3/group (A, B, C, D, E, F, G, H, I, J, K, L, M, N, O, P, Q, R, S, T). **P < 0.01, ***P < 0.001.

**Figure S8.**
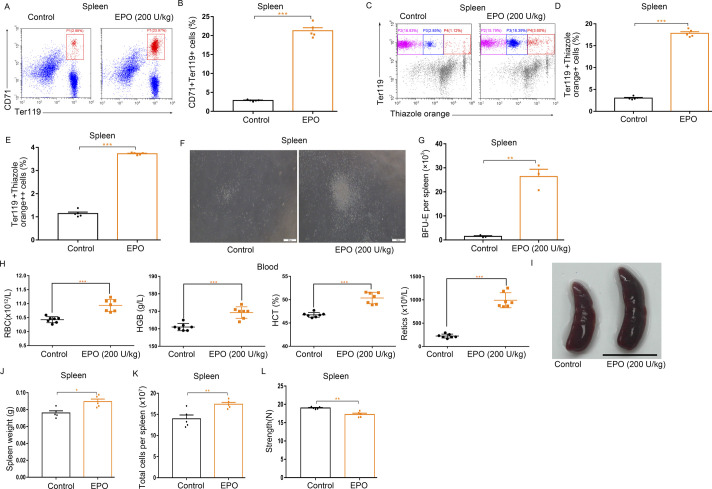
EPO injections significantly enhance splenic erythropoiesis and induce mild splenomegaly. **(A)** Representative flow cytometric plots of CD71^+^Ter119^+^ cells in freshly isolated splenocytes from control and EPO-treated mice. **(B)** Bar graphs showing quantification of CD71^+^Ter119^+^ cells in the spleens of control and EPO-treated mice. **(C)** Representative flow cytometric profiles of Ter119-APC and thiazole orange–stained spleen cells from control and EPO-treated mice. **(D)** Flow cytometric quantification of reticulocytes (Ter119^+^ Thiazole orange^+^) in the spleens from different treatment groups. **(E)** Flow cytometric quantification of erythroblasts (Ter119^+^ Thiazole orange^++^) in the spleens from different treatment groups. **(F)** Representative fields of burst-forming unit erythroid–derived colonies in spleen cultures from control and EPO-treated mice (×100 magnification; scale bar = 200 μm). **(G)** Quantification of the number of burst-forming unit erythroid–derived colonies in spleen cultures from control and EPO-treated mice. **(H)** Peripheral blood hematologic parameters of control and EPO-treated mice. Red blood cell (RBC), hemoglobin (HGB), hematocrit (HCT), and reticulocytes (Retics). **(I)** Representative photographs of spleens from control mice and EPO-treated mice. **(J)** Average weights of spleens from each group. **(K)** Total cell numbers of spleens from each group. **(L)** Compressive strength of spleens from each group. n = 5/group (A, B, C, D, E, I, J, K, L), n = 3/group (F, G), n = 7/group (H). *P < 0.05, **P < 0.01, ***P < 0.001.

**Figure S9.**
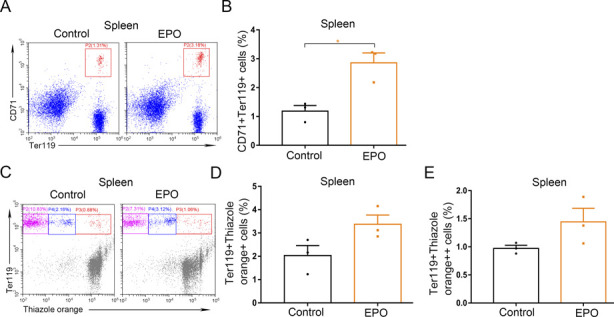
The effects of single dose of EPO (200 U/kg) on spleen. **(A)** Representative flow cytometric plots of CD71^+^Ter119^+^ cells in the spleens of control and EPO-treated mice. **(B)** Bar graphs showing quantification of CD71^+^Ter119^+^ cells in the spleens of control and EPO-treated mice. **(C)** Representative flow cytometric plots of Ter119-APC and thiazole orange-stained splenocytes from control and EPO-treated mice. **(D)** Flow cytometric quantification of reticulocytes (Ter119^+^ Thiazole orange^+^) in the spleens from different treatment groups. **(E)** Flow cytometric quantification of erythroblasts (Ter119^+^ Thiazole orange^++^) in the spleens from different treatment groups. n = 3/group (A, B, C, D, E). *P < 0.05.

Erythropoiesis is driven by EPO that is mainly produced by renal EPO-producing cells in a hypoxia-inducible manner (29). To further study the mechanisms underlying G-CSF-induced erythropoiesis in the spleens of mice, we measured the circulating EPO levels. After G-CSF treatment (50 μg/kg), serum EPO levels in mice increased from 125 to 304 pg/ml (Fig 6A); in keeping with this result, we found that G-CSF treatment resulted in elevated EPO mRNA levels in the kidneys (Fig 6B). EPO is regulated by hypoxia-inducible factor (HIF); likewise, HIF-1α and HIf-2α were found to be up-regulated in the kidneys of mice treated with G-CSF (Fig 6C). Next, we assessed whether EPO is sufficient to induce splenic erythropoiesis. Wild-type mice were given four consecutive daily injections of EPO, after which a robust erythropoietic response was observed to take place in the spleen (Fig S8A–G). Single dose of EPO (200 U/kg) was administered to the mice; the results showed that hyperfunction of splenic erythropoiesis occurred in mice exposed to single dose of EPO (Fig S9).

**Figure S10.**
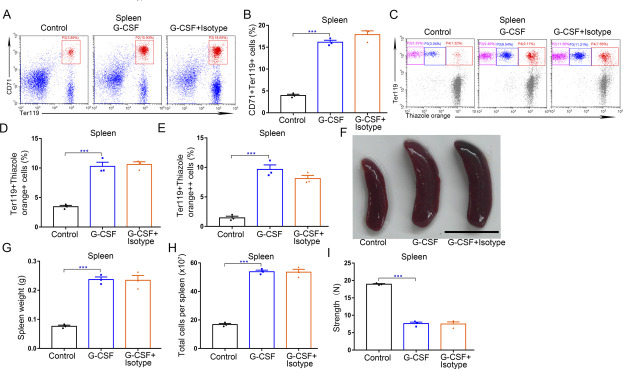
Anti-EPO isotype control has no influence on BM and spleen that changed by G-CSF treatment. **(A)** Representative flow cytometric plots of CD71^+^Ter119^+^ cells in the spleens of control, G-CSF–treated mice, and G-CSF–treated mice with isotype treatment. **(B)** Flow cytometric quantification of CD71^+^Ter119^+^ erythroid cells in the spleens of control, G-CSF–treated mice, and G-CSF–treated mice with isotype treatment. **(C)** Representative flow cytometric profiles of Ter119-APC and thiazole orange–stained cells from the spleens of control, G-CSF–treated mice, and G-CSF–treated mice with isotype treatment. **(D)** Flow cytometric quantification of reticulocytes (Ter119^+^ Thiazole orange^+^) in the spleens of mice. **(E)** Flow cytometric quantification of erythroblasts (Ter119^+^ Thiazole orange^++^) in the spleens of mice. **(F)** Representative images of spleens from WT control mice, G-CSF–treated mice, and G-CSF–treated mice with isotype treatment (scale bar = 1 cm). **(G)** Average weights of spleens from each group. **(H)** Total cell numbers of spleens from each group. **(I)** Compressive strength of spleens from each group. n = 3/group (A, B, C, D, E, F, G, H, I). ***P < 0.001.

Hematologic parameter analysis showed that serial injections of EPO increased red blood cell numbers, hemoglobin levels, hematocrits, and reticulocyte counts in mice (Fig S8H). To certify the cardinal role of EPO in splenic erythropoiesis, mice were treated with G-CSF together with anti-EPO neutralizing antibody (Fig 6D). Anti-EPO treatment reduced the increase in Ter119^+^CD71^+^ cells, reticulocytes, erythroblasts and the numbers of BFU-E in the spleens of mice treated with G-CSF (Figs 6E–K and S10A–E), indicating that neutralization of EPO with antibody inhibits G-CSF–induced splenic erythropoiesis.

**Figure S11.**
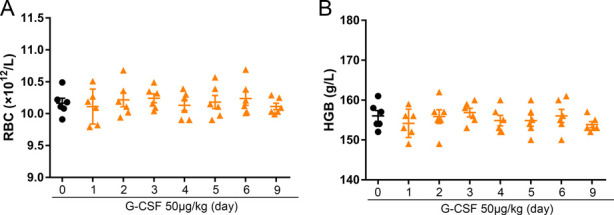
The kinetics of red blood cell numbers and hemoglobin levels in G-CSF–treated mice. **(A)** Peripheral red blood cell (RBC) counts in G-CSF–treated mice. **(B)** Peripheral hemoglobin (HGB) counts in G-CSF–treated mice. n = 6/group (A, B).

In view of the above findings, it was of interest to establish that G-CSF suppressed bone marrow erythropoiesis, thus causing the anemia and renal hypoxia that stimulated secretion of renal EPO that subsequently promoted splenic erythropoiesis. Because red blood cell transfusions can correct anemia and tissue hypoxia (37, 38), we examined the impact of red blood cell transfusions on the response to G-CSF administration (Fig 6L). We transfused G-CSF–treated mice with red blood cells from WT mice and found that there was a significant reduction in serum EPO (Fig 6M) that was accompanied by a significant inhibition of splenic erythropoiesis (Fig 6N–T). We further examined the kinetics of peripheral red blood cell. Evaluation of hematologic parameters revealed that there were no differences in red blood cell numbers and hemoglobin levels between G-CSF-treated mice (on days 1, 2, 3, 4, 5, 6, and 9) and control mice (Fig S11). These results are somewhat contradictory. We speculate that EPO promotes splenic erythropoiesis and rapidly stimulates the release of splenic red blood cells. We further speculate that the oxygen carrying capacity of the red blood cell from splenic erythropoiesis is lower than bone morrow-derived red blood cell.

**Figure 7.**
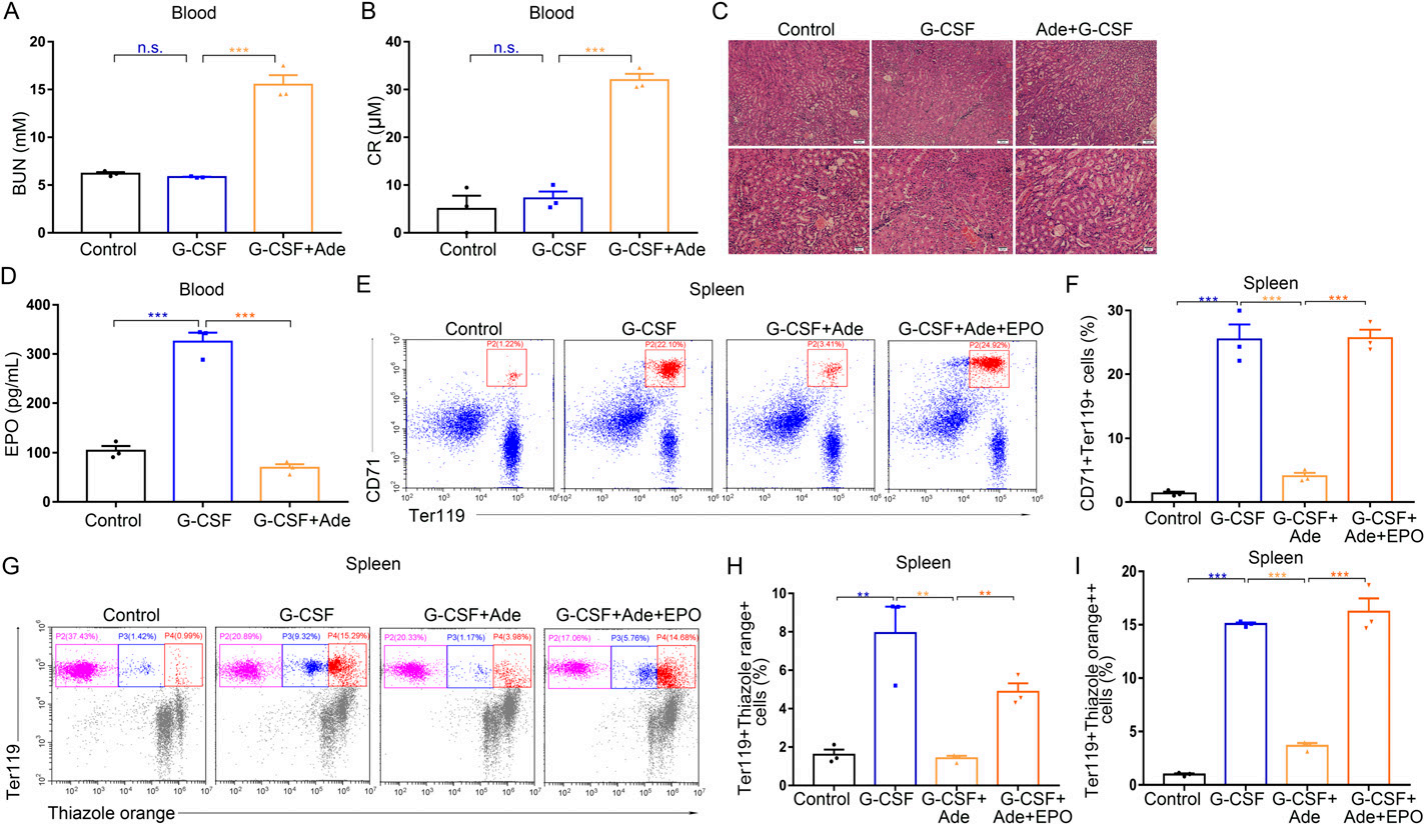
Adenine intake–induced renal dysfunction decreases serum EPO levels and inhibits splenic erythropoiesis in G-CSF–treated mice. **(A, B)** Levels of blood urea nitrogen and serum creatinine in control mice, G-CSF–treated mice, and G-CSF–treated mice fed with adenine. **(C)** Representative histological photomicrographs of kidneys. Original magnification, top panels ×100, and bottom panels ×200. **(D)** Serum levels of EPO in control mice, G-CSF–treated mice, and G-CSF–treated mice fed with adenine. **(E, F, G, H, I)** Flow cytometric analysis of the splenic erythropoiesis in control mice, G-CSF–treated mice, G-CSF–treated mice fed with adenine and G-CSF–treated mice fed with adenine and with EPO treatment. **(E)** Representative flow cytometric plots of CD71^+^Ter119^+^ cells in the spleens. **(F)** Bar graphs showing quantification of CD71^+^Ter119^+^ cells in the spleens from different treatment groups. **(G)** Representative flow cytometric plots of Ter119-APC and thiazole orange–stained splenocytes from different treatment groups. **(H)** Flow cytometric quantification of reticulocytes (Ter119^+^ Thiazole orange^+^) in the spleens from different treatment groups. **(I)** Flow cytometric quantification of erythroblasts (Ter119^+^ Thiazole orange^++^) in the spleens from different treatment groups. n = 3/group (A, B, C, D, E, F, G, H, I). **P < 0.01, ***P < 0.001, n.s., no significance.

Adenine intake in mice induces progressive kidney damage (39). To acquire greater insight into the key role of the kidneys in G-CSF–induced splenic erythropoiesis, we used a model of adenine-induced chronic kidney injury. Treatment of mice with a 0.3% adenine diet induced continuous progressive kidney damage with increased serum urea and creatinine (Fig 7A and B). Renal histology revealed tubular atrophy, erosion of proximal tubular brush borders with flattening of the epithelium, and focal tubular epithelial hypertrophy (Fig 7C). As expected, adenine-induced chronic kidney injury resulted in lower serum EPO levels (Fig 7D) and inhibited splenic erythropoiesis in mice treated with G-CSF (Fig 7E–I). High-dose EPO injection rescued the erythropoietic function of spleens under kidney injury conditions in G-CSF–treated mice (Fig 7E–I). Taken together, these results indicate that renal EPO plays a key role in G-CSF–induced erythropoiesis.

### G-CSF treatment results in dose-dependent splenomegaly that is associated with increased fragility of spleen in G-CSF–treated mice

**Figure 8.**
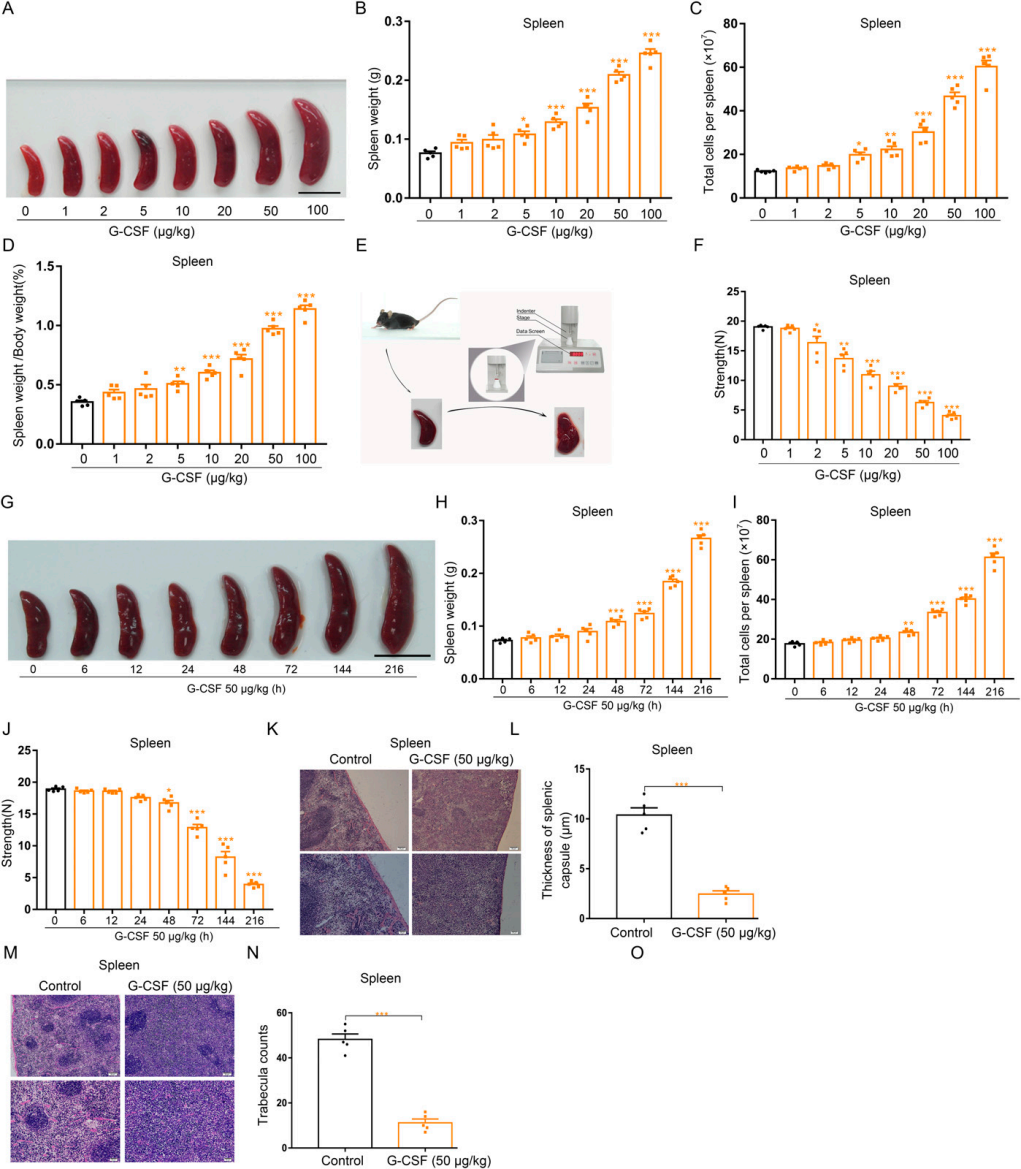
G-CSF treatment leads to splenomegaly and increases splenic fragility in mice. **(A)** Macroscopic views of spleens in control and G-CSF–treated mice (scale bar = 1 cm). **(B)** Average weights of spleens in control and G-CSF–treated mice. **(C)** Total cell numbers of spleens in control and G-CSF–treated mice. **(D)** The ratios of spleen weight with respect to body weight. **(E)** Schema showing the compression test of spleen. **(F)** Compressive strength of spleens from each group. **(G)** Representative images of spleens from control and G-CSF-treated mice (scale bar = 1 cm). **(H)** Average weights of spleens from each group. **(I)** Total cell numbers of spleens from each group. **(J)** Compressive strength of spleens from each group. **(K, M)** Histopathological views of spleen tissues from the WT control mice and G-CSF–treated mice. Splenic sections were stained with H&E. The thickness of splenic capsule was measured under the microscope and the numbers of trabeculae were counted in histological photomicrographs. Original magnification, upper panels ×100 and lower panels ×200. **(L)** Quantification of thicknesses of splenic capsules. **(N)** Quantification of the numbers of trabecula in different groups (×100). n = 5/group (A, B, C, D, E, F, G, H, I, J, K, L, M, N). *P < 0.05, **P < 0.01, ***P < 0.001.

**Figure S12.**
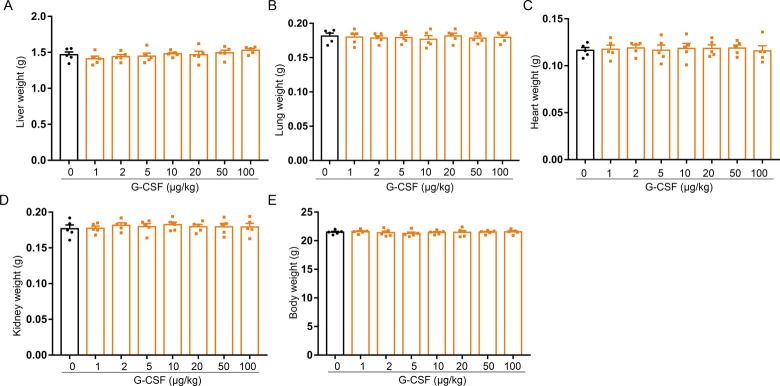
G-CSF treatment has no influence on the liver, lung, heart, kidney, and body weight. **(A)** Liver weights of control and G-CSF–treated mice. **(B)** Lung weights of control and G-CSF–treated mice. **(C)** Heart weights of control and G-CSF–treated mice. **(D)** Kidney weights of control and G-CSF–treated mice. **(E)** Body weights of control and G-CSF–treated mice. n = 5/group (A, B, C, D, E).

Spleen is a fragile organ; severe splenomegaly may increase the risk for splenic rupture that is a potentially serious event (40, 41). To confirm the correlation between splenomegaly and splenic fragility in G-CSF–treated mice, we conducted a dose-finding experiment. We observed that G-CSF–treated mice developed progressively spleen enlargement with the incremental dosage of G-CSF administration (Fig 8A). Measurement of weight and cell number revealed significant increases in both splenic weights and splenocytes in mice treated with G-CSF from 5 to 100 μg/kg (Fig 8B and C); spleen/body weight ratios also increased in G-CSF–treated mice relative to control mice (Fig 8D). By contrast, there was no increase in the liver, lung, heart, kidney, or body weight in animals receiving G-CSF treatment (Fig S12). In an attempt to evaluate the fragility of spleen, we carried out a compression test on spleen (Fig 8E). The compressive strength (Fig 8F) from the splenic test indicated that G-CSF–treated mice underwent a progressive increase in splenic brittleness proportional to the increase in G-CSF dose. G-CSF–induced splenomegaly and increase of splenic brittleness were also time-dependent (Fig 8G–J). Single dose of G-CSF did not induced splenomegaly (Fig S2P–S). The spleen is surrounded by a capsule that contains collagen, elastic fibers, and smooth muscle and provides some rigidity to the spleen together with trabeculae that protrudes from capsule into splenic tissue (42, 43); rapid splenic enlargement can produce a thin and tense splenic capsule that is susceptible to rupture (44, 45). Therefore, we measured the thickness of splenic capsules by histology and found that the mean thickness of splenic capsules in G-CSF–treated mice (50 μg/kg) was 2.46 μm compared with 10.38 μm in control mice (Fig 8K and L). The thin capsule was accompanied by fewer and smaller trabeculae in G-CSF-treated mice (Fig 8M and N). The decreased thickness of splenic capsule and lack of splenic trabeculae may account for the increased fragility of spleens from G-CSF-treated mice.

### TLR4-mediated G-CSF production is essential for inflammation-associated splenomegaly in mice

**Figure S13.**
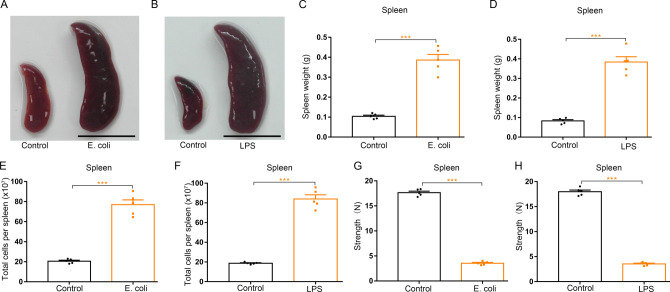
Either *E. coli* infection or LPS treatment induces splenomegaly and high fragility of spleen in mice. **(A)** Representative images of spleens from control mice and *E. coli*-infected mice (scale bar = 1 cm). **(B)** Representative images of spleens from control mice and LPS-treated mice (scale bar = 1 cm). **(C)** Average weights of spleens from control mice and *E. coli*–infected mice. **(D)** Average weights of spleens from control mice and LPS-treated mice. **(E)** Total cell numbers of spleens from control mice and *E. coli*–infected mice. **(F)** Total cell numbers of spleens from control mice and LPS-treated mice. **(G)** Compressive strength of spleens from control mice and *E. coli*–infected mice. **(H)** Compressive strength of spleens from control mice and LPS-treated mice. n = 5/group (A, B, C, D, E, F, G, H). ***P < 0.001.

**Figure 9.**
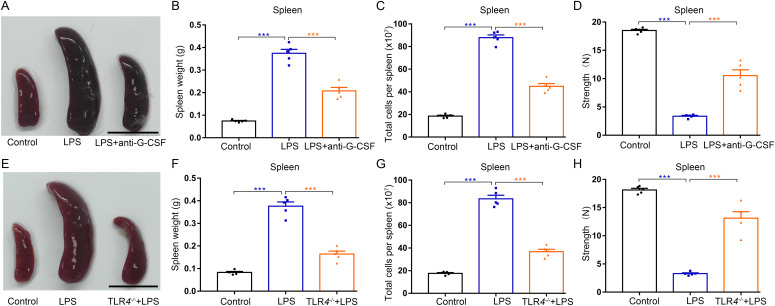
*TLR4*-mediated G-CSF production contributes to splenomegaly and high fragility of spleen during systemic inflammation in mice. **(A)** Representative images of spleens from WT control mice, LPS-treated mice, and LPS-treated mice with anti–G-CSF treatment (scale bar = 1 cm). **(B)** Average weights of spleens from each group. **(C)** Total cell numbers of spleens from each group. **(D)** Compressive strength of spleens from each group. **(E)** Representative images of spleens from WT control mice, LPS-treated mice, and *TLR4*^*−/−*^ mice treated with LPS (scale bar = 1 cm). **(F)** Average weights of spleens from each group. **(G)** Total cell numbers of spleens from each group. **(H)** Compressive strength of spleens from each group. n = 5/group (A, B, C, D, E, F, G, H). ***P < 0.001.

Next, we tested to determine the pathological relevance of G-CSF in mice with splenomegaly during systemic inflammatory processes. Macroscopic analysis revealed that both *E. coli*-infected mice and LPS-treated mice developed spleen enlargement (Fig S13A and B). Comparison of weights and cell counts showed significant increases in splenic weight and splenocyte number of mice infected with *E. coli* or treated with LPS (Fig S13C–F). Both infection of mice with *E. coli* and treatment of mice with LPS characteristically decreased the compressive strength of the spleens (Fig S13G and H), indicating that mice bearing infection-related systemic inflammation undergo a progressive increase in splenic brittleness. The enhancements in spleen size, splenic weight, and splenocyte number were ameliorated by continuous neutralization of G-CSF in LPS-treated mice; importantly G-CSF neutralization decreased the splenic friability in LPS-treated mice (Figs 9A–D and S7O–R). Next, we investigated the potential impact of TLR4 on LPS-induced splenomegaly and observed that TLR4 deficiency almost prevented splenomegaly and increased splenic friability in LPS-treated mice (Fig 9E–H). Single treatment of LPS and single infection of *E. coli* were not associated with splenomegaly (Figs S5O–R and S6O–R). Together, our findings suggest that G-CSF mediates inflammation-induced splenomegaly.

### Splenic erythropoiesis partially contributes to G-CSF–induced splenomegaly and high splenic fragility in mice

**Figure 10.**
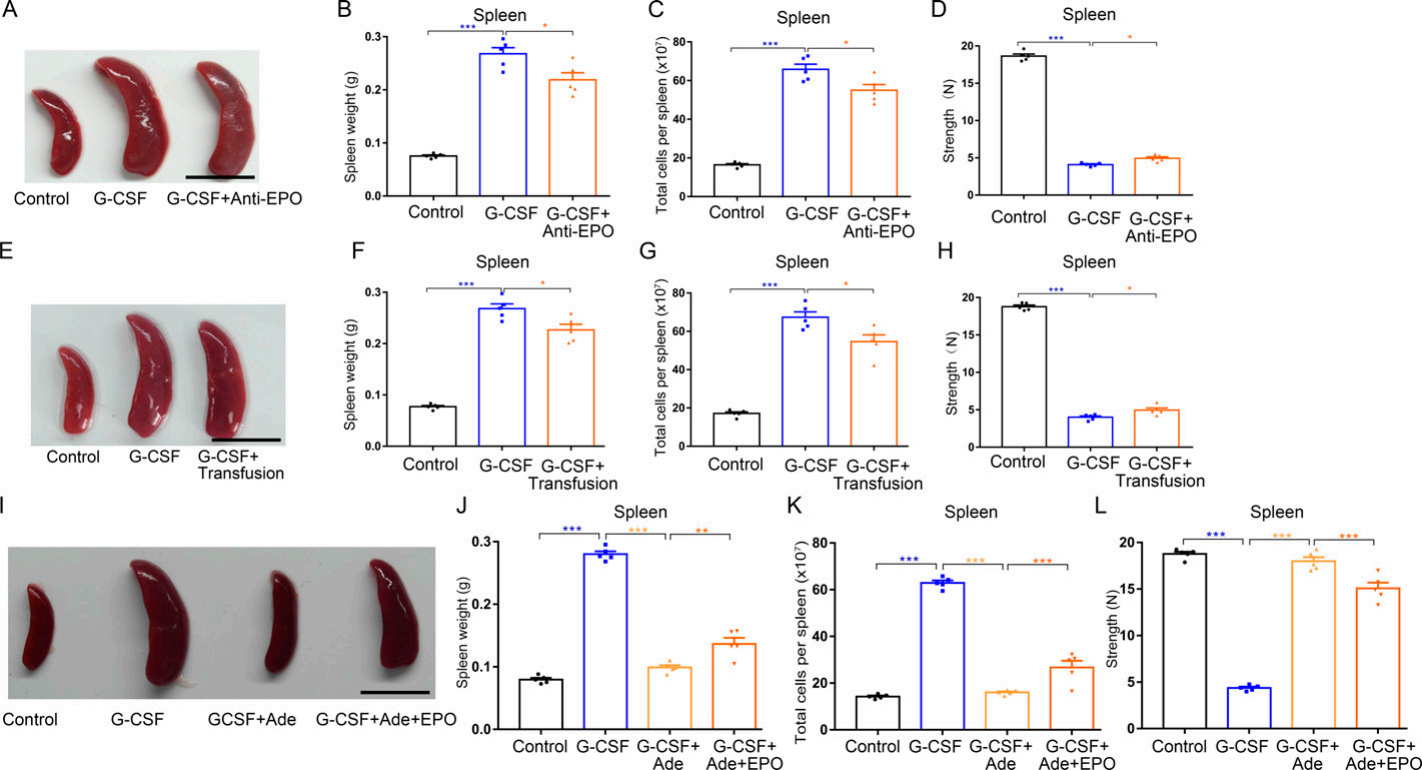
EPO neutralization, red blood cell transfusion, and adenine-induced kidney injury alleviate splenomegaly in G-CSF–treated mice. **(A)** Representative images of spleens from WT control mice, G-CSF–treated mice, and G-CSF–treated mice with anti-EPO treatment (scale bar = 1 cm). **(B)** Average weights of spleens from each group. **(C)** Total cell numbers of spleens from each group. **(D)** Compressive strength of spleens from each group. **(E)** Representative images of spleens from WT control mice, G-CSF–treated mice, and G-CSF–treated mice with red blood cell transfusion (scale bar = 1 cm). **(F)** Average weights of spleens from each group. **(G)** Total cell numbers of spleens from each group. **(H)** Compressive strength of spleens from each group. **(I)** Representative images of spleens from control mice, G-CSF–treated mice, G-CSF–treated mice fed with adenine, and G-CSF–treated mice fed with adenine and with EPO treatment (scale bar = 1 cm). **(J)** Average weights of spleens from each group. **(K)** Total cell numbers of spleens from each group. **(L)** Compressive strength of spleens from each group. n = 5/group (A, B, C, D, E, F, G, H, I, J, K, L). *P < 0.05, **P < 0.01, ***P < 0.001.

Splenomegaly is a complication of splenic erythropoiesis (34, 46). Next, we sought to address potential roles of splenic erythropoiesis in G-CSF–induced splenomegaly and high fragility of spleen. We first treated mice with G-CSF together with anti-EPO neutralizing antibody, and we found that neutralization of EPO only slightly inhibited the progression of splenic enlargement (Figs 10A–C and S10F–H) and mildly increased the compressive strength of spleens (Figs 10D and S10I). These results indicate that EPO neutralization alleviates but does not reverse splenomegaly and high fragility of spleen induced by G-CSF. Similarly, red blood cell transfusions slightly mitigated G-CSF-induced splenomegaly and fragility rupture of spleen (Fig 10E–H). To further validate the role of EPO in splenomegaly and splenic fragility, we treated WT mice with EPO and found that the splenic weight, splenocyte number, and splenic fragility mildly increased after daily injection of EPO (200 U) for four consecutive days (Fig S8I–L). We then investigated the effects of kidney injury on G-CSF-induced splenic enlargement and fragility rupture. In contrast to EPO neutralization and red blood cell transfusion, adenine-induced kidney injury almost blocked G-CSF–associated splenomegaly and significantly increased the compressive strength of spleens in mice treated with G-CSF (Fig 10I–L). To unequivocally determine the role of splenic erythropoiesis in G-CSF–induced splenomegaly and fragility rupture of spleen, we monitored the effects of high-dose EPO injections on the spleens in G-CSF–treated mice with kidney injury. The spleen weight, splenocyte number, and splenic fragility in mice with kidney injury treated with G-CSF moderately increased after high-dose EPO administration (Fig 10I–L). These results indicate that splenic erythropoiesis partially accounts for G-CSF–induced splenomegaly and splenic fragility.

## Discussion

Bone marrow erythropoiesis dysfunction and anemia accompany many types of inflammation, particularly the inflammation caused by bacterial pathogens (14, 15, 16, 17, 18). Inflammation often stimulates splenic erythropoiesis; the lack of mechanistic explanations for inflammation-associated splenic erythropoiesis precludes thorough assessment of its impact on human health. G-CSF can be produced by a variety of cell types following appreciate stimulation, including monocytes/macrophages, vascular endothelial cells, and fibroblasts (47). After in vivo LPS or *E. coli* stimulation, endothelial cells are the prime sources of G-CSF. Inflammatory responses are often triggered by TLR4 that translates pathogen signals into G-CSF production (4). Our data extend this knowledge by demonstrating that G-CSF mediates the repression of bone marrow erythropoiesis and enhancement of splenic erythropoiesis in mice during systemic inflammation.

All cell types in the circulation and some cells in tissues of the body are derived from hematopoietic stem cells. Both erythropoiesis and granulopoiesis are complex processes in which multipotential hematopoietic stem cells proliferate, differentiate, and eventually form mature erythrocytes and granulocytes. Bone marrow is the primary erythropoietic and granulopoietic organ, where myeloid progenitor cells make all myeloid cells. As common myeloid progenitors mature, they differentiate into precursors for either erythrocytes and megakaryocytes or granulocytes and monocytes, but not both (48, 49). We found that G-CSF has opposite effects on granulopoiesis and erythropoiesis in bone marrow, indicating that some amount of competition exists in differentiation cascade of granulopoiesis and erythropoiesis. The competition between granulopoiesis and erythropoiesis in the presence of inflammation has been reported by several groups (17, 18, 50). Further research on this topic will open up new horizons for understanding the pathogenesis of erythropoiesis dysfunction and will identify potential new therapy strategies for treating anemia of inflammation.

Splenic erythropoiesis becomes important during repression of bone marrow erythropoiesis (11, 12). Regulation of physiologic and pathologic erythropoiesis is dependent on EPO that is generated principally in the kidney. We demonstrated that G-CSF promotes splenic erythropoiesis by elevating EPO levels that are regulated by HIF activity in response to low oxygen tension.

Splenomegaly has become increasingly recognized as one of the most important issues in health care and health outcomes and it is of particular importance in patients who are undergoing treatment with G-CSF or experiencing a microbial infection. We demonstrate, for the first time to our knowledge, that splenomegaly increases the fragility of spleen by reducing splenic trabecula and the thickness of capsule in G-CSF–treated mice. Although the prevailing view is that splenomegaly accompanies the splenic erythropoiesis, our results show that splenic erythropoiesis makes only a partial contribution to G-CSF–induced splenomegaly and splenic fragility. We then investigated the contribution neutrophil on G-CSF–induced splenomegaly. There were low levels of neutrophils in the spleen of control mice, whereas G-CSF administration led to the emergence of distinguishable neutrophils that gradually increased after the incremental doses of G-CSF (data not shown). We postulate that the ingress of bone marrow-derived neutrophils into spleen contributes the G-CSF–induced splenomegaly and high splenic fragility. Further studies should address the main causes of G-CSF–induced splenomegaly.

## Conclusions

Our data indicate that TLR4 initiates G-CSF up-regulation that suppresses bone marrow erythropoiesis under the circumstances of systemic inflammation. G-CSF–induced repression of bone marrow erythropoiesis leads to anemia and hypoxia that stimulates the expression of erythropoietin by renal cells, which in turn enhances splenic erythropoiesis. Inhibition of G-CSF signaling pathway may be potential strategy to prevent inflammation-associated dysfunction of erythropoiesis and treat anemia of inflammation.

## Materials and Methods

### Mice

All animal experiments were approved and conducted in full accordance with protocols approved by the Ethical Committee of School of Basic Medical Sciences, Shandong University. C57BL/6 mice were purchased from the laboratory animal center of Shandong University. *TLR4*^−/−^ mice (C57BL/10ScNJ *Mus musculus*, Cat. no. JAX:003752; Jackson Laboratory) were purchased from the Jackson laboratory. Mice were maintained in a specific pathogen-free facility at Shandong University on a 12 h/12 h light/dark cycle with free access to food and water. 8- to 10-wk-old male mice were used in all animal experiments.

### G-CSF treatment

To assess the effects of G-CSF on spleen and bone marrow, mice were subcutaneously injected twice daily with different doses (1, 2, 5, 10, 20, 50, or 100 μg/kg) of G-CSF (Cat. no. S19990049; Qilu Pharmaceutical) for nine consecutive days. Mice were weighed and euthanized on day 9, and their spleens, femurs, and other organs were collected. The femurs were photographed, and then the bone marrow cells were collected, counted, and processed for flow cytometry. The spleens were photographed, weighted and fixed for standard hematoxylin and eosin (H&E) staining, and the splenocytes were collected, counted, and prepared for flow cytometry. The livers, lungs, hearts, and kidneys were weighted.

### Compressive strength test

Compressive strength of spleen was measured using a mechanical property testing machine (Jinan Yiyan Science and Technology Development Co., Ltd.) with a load cell. Microcomputer pressure feedback control technology was used in the testing machine to make the pressure and downward velocity become linear inverse ratio, and a very uniform pressure was obtained in the whole pressure process, which avoided the error caused by impact pressure. The experiment was conducted at ambient temperature and the strength was recorded when the spleen was crushed.

### Histological analysis

Spleens were fixed in 4% formaldehyde (vol/vol) in PBS for 48 h, embedded in paraffin, cut into 5-μm sections, stained with H&E, and evaluated using light microscopy. The splenic capsule thickness and trabecula were evaluated by two pathologists in a blinded fashion.

### Flow cytometric analysis

Bone marrow cells were flushed out of femurs. Splenocytes were isolated by mechanical dissociation of the spleen. Flow cytometric analysis of erythroid cells using double staining with Ter119 and CD71 antibodies was performed as described (32). Cells were washed with PBS, and 1 × 10^6^ cells from bone marrow and spleen were incubated with 0.1 mg/ml rat IgG (Cat. no. SP032; Solarbio) for 10 min at 4°C. Cells were then incubated with APC-conjugated anti-mouse Ter119 (Cat. no. 557909; BD Biosciences) and PE-labeled anti-mouse CD71 (Cat. no. 553267; BD Biosciences) for 30 min. Flow cytometric analysis of reticulocytes and erythroblasts using double staining with thiazole orange and anti-Ter119-APC was performed as described (32). Cells were stained with 0.2 μg/ml thaizole orange (Cat. no. 390062; Sigma-Aldrich) at room temperature for 20 min. Cells were washed, blocked with rat IgG for 10 min at 4°C, and then incubated with APC-conjugated anti-mouse Ter119 for 30 min. After staining, the cells were washed with PBS, and the samples were analyzed on a Beckman Cytoflex FCM using CytExpert software. For GATA-1 analysis, the cells were fixed in 4% paraformaldehyde, permeabilized with ice-cold 90% methanol, incubated with rabbit IgG, and stained with PE-conjugated anti–GATA-1 (Cat. no. 13353; Cell Signaling Technology). Stained cells were washed with PBS and analyzed by flow cytometry.

### Hematological parameters

Mice were anesthetized with sodium pentobarbital. Blood was extracted by cardiac puncture and collected into EDTA-coated tubes. Complete blood counts were determined using an ADVIA 2120i Hematology Analyzer (Siemens).

### Wright–Giemsa staining

The peripheral blood and bone marrow cells of mice were harvested after 9 d of G-CSF treatment. The cells were washed, resuspended in PBS, smeared on slides, stained with Wright–Giemsa (Cat. no. CD005; MACGENE), and photographed under a microscope.

### CFU-GM colony assay

CFU-GM colony assay was performed as previously described (51). Bone marrow cells (1 × 10^4^) from control and G-CSF–treated mice were seeded into methylcellulose-based media containing CFU-GM–promoting growth factors (Cat. no.:03534; Stem Cell Technologies) and incubated for 10 d at 37°C in 5% CO_2_.

### BFU-E colony assay

BFU-E cells were analyzed as previously reported (13). Bone marrow or spleen cells from control and G-CSF–treated mice were sorted, plated in methylcellulose (Cat. no.:3436; Stem Cell Technologies), and incubated for 10 d at 37°C in 5% CO_2_.

### Splenectomy

Splenectomy was performed as previously described (13). Mice were placed in a warm environment and anesthetized with sodium pentobarbital. The skin covering the spleen was prepared for splenectomy by depilation and sterilization. After opening the dorsal left flank, the spleen was gently lifted and a 2-0 silk suture tie was placed underneath the spleen as proximal to the origin of the splenic vessels as possible. Subsequently, the spleen was gently resected from the connective tissue. The peritoneum was then closed with a running 4-0 absorbable silk suture, and the skin was re-approximated with a running 4-0 silk suture. In the sham-operated group, the same operation was performed without removing the spleens from the mice. After the operation, the mice recovered for at least 2 wk before the onset of experiments.

### Measurement of serum cytokine concentration

Blood was obtained from mice via cardiac puncture, clotted at 4°C for 2 h, and then centrifuged at 3,000*g* for 15 min. Serum was collected in a sterile tube. Serum EPO concentrations were determined using an ELISA kit (Cat. no. MEP00B; R&D Biosystems) according to the manufacturer’s instructions. Serum G-CSF concentrations were measured by flow cytometry using the mouse G-CSF Flex-Set bead array (Cat. no. 560152; BD Biosciences), which was used according to the manufacturer’s instructions.

### Quantitative RT-PCR

Total RNA was isolated with TRIzol reagent from kidneys, according to the manufacturer’s instructions. cDNA was synthesized from 2 μg total RNA using a High Capacity RNA-tocDNA kit (TIANGEN). EPO expression was analyzed using qRT-PCR on a CFX96 Real-Time System with SYBR GREEN and normalized to β-actin. The PCR protocol was 95°C for 15 min, followed by 40 cycles of 95°C for 10 s and 60°C for 32 s.

### Western blotting

Kidney protein lysates were collected in radio immunoprecipitation assay (RIPA) buffer supplemented with complete protease inhibitor cocktail using a tissue homogenizer. Insoluble debris was removed by centrifugation at 12,000*g* at 4°C for 15 min, and the supernatant was transferred to a clean polypropylene tube. An equal volume of sample loading buffer was added and the tubes were capped tightly, vortexed briefly, and boiled for 10 min. Lysates were resolved by SDS–PAGE and transferred to polyvinylidene fluoride (PVDF) membranes via electroblotting. Membranes were incubated in 5% non-fat milk for 1 h to block non-specific binding and incubated overnight at 4°C with specific primary antibodies (anti-HIF-1α, from Abcam, Cat. no. ab16066; anti-HIF-2α, from Abways, Cat. no. CY5098). The membranes were probed with secondary antibodies at room temperature for 1 h and then incubated with ECL-Plus (Cat. no. WBKLS0100; Millipore) as directed by the manufacturer. Western blots were performed on scanned films using ImageJ software.

### In vivo EPO neutralization

Mice were subcutaneously injected with 50 μg/kg G-CSF twice daily on days 0–9. Rat anti-EPO antibody (10 μg per mouse, Cat. no. MAB959; RD Systems) or rat IgG2A isotype control (Cat. no. MAB006; RD Systems) in 200 μl PBS were administered on days 2, 5, and 8 via intraperitoneal injection.

### Murine red blood cell collection and transfusion

Mice were anesthetized with sodium pentobarbital. Blood was collected by cardiac puncture into vacutainer tubes containing EDTA-K_2_ (3.6 mg). Red blood cells were isolated by centrifugation (1,500*g* for 10 min), and the plasma and buffy coat were removed. Red blood cells were washed three times with PBS to remove residual EDTA. After G-CSF injection, recipient mice were transfused with 150 μl (equivalent of 150 μl blood) red blood cells on days 2, 4, 6, and 8 via tail vein injection. The control mice were injected with PBS.

### EPO treatment

To assess the effect of EPO on splenic erythropoiesis, mice were subcutaneously injected once a day with EPO (200 U/kg, 3SBIOC, Cat. no. S20010001) or saline for four consecutive days.

### Adenine treatment

For the model of kidney injury, mice were fed with a 0.3% adenine diet for 15 d. Mice were subcutaneously injected with 50 μg/kg G-CSF twice daily on days 7–15. To provide a 0.3% adenine-containing chow consumed by the mice, adenine was mixed with a 20% casein-based diet that blunted the smell and taste. The mice of control group were fed with the same 20% casein diet as the 0.3% adenine group without the addition of adenine. For EPO treatment, mice were subcutaneously injected twice a day with EPO (1,000 U/kg) on days 7–15.

### Mouse model of *E. coli*- or LPS-induced systemic inflammation

For systemic infection, mice were intraperitoneally injected with 1 × 10^6^ *E. coli* (Cat. no. CW0808S; CWBIO) once every 2 d for 9 d. For LPS-induced systemic inflammation, WT and *TLR4*^*−/−*^ mice were intraperitoneally injected with 0.5 mg/kg LPS (Cat. no. L2880; Sigma-Aldrich) twice daily for 9 d. To neutralize G-CSF, LPS-treated mice were intravenously injected with anti–G-CSF neutralizing antibody (Cat. no. MAB414; RD Systems) or rat IgG1 isotype control (Cat. no. MAB005; RD Systems) on days 2, 5, and 8 during LPS administration.

### Statistical analysis

All data are pooled from at least three independent experiments. Data analysis was performed using GraphPad Prism. Statistical analyses were performed using unpaired *t* tests or one-way ANOVA. Data are presented as mean ± SEM. *P*-values less than 0.05 were considered statistically significant.

## Supplementary Material

Reviewer comments
